# A cyclical switch of gametogenic pathways in hybrids depends on the ploidy level

**DOI:** 10.1038/s42003-024-05948-6

**Published:** 2024-04-08

**Authors:** Dmitrij Dedukh, Anatolie Marta, Ra-Yeon Myung, Myeong-Hun Ko, Da-Song Choi, Yong-Jin Won, Karel Janko

**Affiliations:** 1 Laboratory of Non-Mendelian Evolution, Institute of Animal Physiology and Genetics of the CAS, Liběchov, Czech Republic; 2 Division of EcoScience, Ewha Womans University, Seoul, South Korea; 3 Kosoo Ecology Institute, Seoul, South Korea; 4 Department of Biology and Ecology, Faculty of Science, University of Ostrava, Ostrava, Czech Republic

**Keywords:** Cytogenetics, Evolutionary biology, Germline development

## Abstract

The cellular and molecular mechanisms governing sexual reproduction are conserved across eukaryotes. Nevertheless, hybridization can disrupt these mechanisms, leading to asexual reproduction, often accompanied by polyploidy. In this study, we investigate how ploidy level and ratio of parental genomes in hybrids affect their reproductive mode. We analyze the gametogenesis of sexual species and their diploid and triploid hybrids from the freshwater fish family Cobitidae, using newly developed cytogenetic markers. We find that diploid hybrid females possess oogonia and oocytes with original (diploid) and duplicated (tetraploid) ploidy. Diploid oocytes cannot progress beyond pachytene due to aberrant pairing. However, tetraploid oocytes, which emerge after premeiotic genome endoreplication, exhibit normal pairing and result in diploid gametes. Triploid hybrid females possess diploid, triploid, and haploid oogonia and oocytes. Triploid and haploid oocytes cannot progress beyond pachytene checkpoint due to aberrant chromosome pairing, while diploid oocytes have normal pairing in meiosis, resulting in haploid gametes. Diploid oocytes emerge after premeiotic elimination of a single-copied genome. Triploid hybrid males are sterile due to aberrant pairing and the failure of chromosomal segregation during meiotic divisions. Thus, changes in ploidy and genome dosage may lead to cyclical alteration of gametogenic pathways in hybrids.

## Abstract (web summary)

The cytogenetic analysis of gonial cells and meiocytes on Korean loach complex suggest that instant switch between sexual to asexual reproduction in hybrids depend on their ploidy and genome dosage.

## Introduction

Sexual reproduction is the ubiquitous feature of eukaryotes and includes the meiotic formation of reduced gametes, fertilization, and development of new organisms^1,2^. While the underlying cellular and molecular machinery is primarily conserved^1^, interspecific hybridization may disrupt the usual reproductive pathways. Such a disruption often reduces fertility in hybrids^3–5^. Nevertheless, hybridization may also lead to the emergence of novel traits, including various forms of asexual reproduction^6–10^. Forms of asexual reproduction have traditionally been categorized based on types of produced gametes and whether sperm is, or is not, required for their development^7,9,10^. Among these, parthenogenesis refers to the production of unreduced (clonal) gametes, which spontaneously develop into clonal progeny^7,9,10^. Gynogenesis (or sperm-dependent parthenogenesis) also involves the production of unreduced (clonal) gametes, but they require sperm to trigger the development without karyogamy^7,9–11^. Kleptogenesis and hybridogenesis are referred to as hemi- or mero-clonal reproduction modes, where part of an asexual’s genome is eliminated, while the other part is passed to gametes requiring fertilization with karyogamy to restore the diploidy^7,9–12^.

**Fig. 1:**
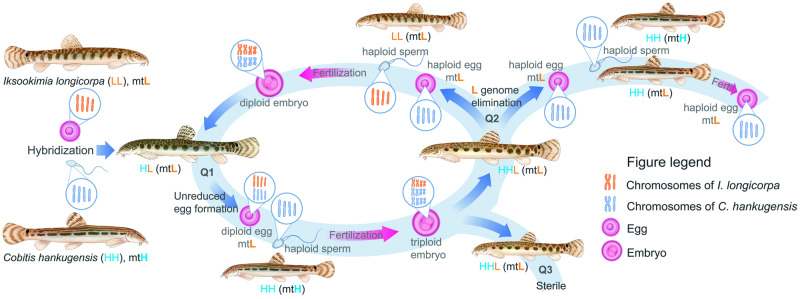
Schematic overview of gametogenesis and reproduction of diploid and triploid hybrids within *C. hankugensis-I. longicorpa* complex (redrawn from refs. ^38,41,44^). After crosses of two parental sexual species, *Cobitis hankugensis* (HH, marked in blue) and *Iksookimia longicorpa* (LL, marked in orange), diploid hybrids (HL) are produced with the mitochondrial DNA (designated as ‘mt’) from one of the sexual species (L). Diploid hybrids form diploid clonal gametes with ‘L’ mtDNA. After fertilization of such eggs by sperm of one of the parental species, triploid hybrids with L mtDNA appear (HHL). In triploids, a single-copied genome (L) is eliminated during their gametogenesis, and the remaining haploid gametes (HH) produce haploid ‘H’ gametes with ‘L’ mtDNA. After fertilization of such gametes by sperm from *C. hankugensis*, diploid sexual species appear but with ‘L’ mtDNA. After fertilization of gametes produced by triploids by sperm from the other parental species, *I. longicorpa*, new diploid hybrids appear with ‘L’ mtDNA. Q1-3 indicates the gap of knowledge in studied asexual hybrid complex: Q1. The cytogenetic mechanisms underlying unreduced gametes formation by diploid hybrids; Q2. Gametogenic stage and mechanisms of putative genome elimination in triploid hybrid females; Q3. Gametogenic alteration underlying hybrid sterility in triploid hybrid males.

Such a classification of asexual reproductive modes suggests that fundamentally different cellular and molecular mechanisms may yield the identical patterns of genome transmission^10,11^. For instance, the formation of clonal gametes may result from several distinct mechanisms. Clonality may be achieved by achiasmatic meiosis, as observed in hybrid *Poecilia formosa*, which maintains oogonia ploidy unchanged but features univalents formation during the first meiotic prophase without pairing and recombination^13,14^. Hypothetically, the reductional division is skipped here, but the equational division is preserved, resulting in clonal progeny^13,14^. An alternative mechanism leading to clonality involves the premeiotic genome endoreplication (Fig. 1), which leads to an increase ploidy in oogonial cells^13,15–22^. Such oogonia with duplicated genomes undergo meiosis, with chromosomal pairing limited to duplicated copies of the same chromosomes, resulting in offspring with no genetic variability^16,17,19–21,23^.

Interestingly, several types of gametogenic programs may occur simultaneous within the same individual. In clonal hybrids of European spined loaches (Cobitidae), most oocytes enter meiosis non-duplicated and face problems in pairing of orthologous chromosomes^24^. Endoreplication occurs in only a minor proportion of gonial cells (1–6%) but rescues their fertility by ensuring proper bivalent formation between identical chromosomal copies^24^. Such a crosslink between hybrid sterility and clonality led Janko and co-authors (2018)^8^ and Stöck and co-authors (2021)^10^ to propose hybrid asexuality as a specific type of postzygotic reproductive isolation mechanism. Still, the generality of this process remains to be tested (see also^25^).

After producing clonal eggs, asexuals have to avoid fertilization to prevent ploidy elevation. To maintain (hemi-)clonal lineages in natural populations, hybridogenetic and gynogenetic organisms often exploit males of sexual species as sperm donors without incorporating their genetic material^26–30^. Occasionally, failure to eliminate the sperm pronucleus from the zygote can result in the emergence of triploid offspring^31–33^. It is generally assumed that such triploids retain the same type of gynogenetic reproductive mode as their diploid hybrid ancestors^12,14,17,20,24,34^. However, variability exists as some triploids may switch reproductive modes and perform genome elimination during gametogenesis^15,31,35–38^. For instance, in *Squalius, Phoxinus, Cobitis-Iksookimia*, and *Misgurnus* hybrid complexes, a single-copied genome (for example, AAB hybrids between parental species A and B eliminate the genome B) is eliminated. In contrast, the double-copied genomes (AA in AAB hybrids) undergo meiosis with pairing and recombination, yielding haploid gametes^31,36–40^. Such gametogenic alteration is called triploid or meiotic hybridogenesis^31^.

These examples above illustrate how dynamic the transitions between sexual and asexual modes (and back) may be, often being influenced by the ploidy level of the individuals. We emphasize, though, that gametogenic alterations were investigated only in a limited number of asexual taxa, while in the vast majority of asexuals, they are either hypothetic or unknown. In addition, the scenarios mentioned above, assuming the genome elimination is mainly based on genetic analysis of offspring from crosses of triploid animals with their parents, have not been tested by detailed analysis of meiotic and premeiotic processes. In particular, the cellular mechanisms underlying switches in reproductive modes between diploid and triploid hybrids, and ensuring the genome elimination in triploid hybrids remain unknown^36–40^. This lack of information and experiment may pose severe problems in understanding how reproductive pathways alter in response to hybridization and polyploidy since, as we discussed above, the same type of allelic inheritance may be caused by fundamentally different gametogenic mechanisms.

A suitable model to address such questions in detail is the *Cobitis hankugensis-Iksookimia longicorpa* hybrid complex of Korean loaches^37,38,41–43^. In this complex (formerly reported as *C. sinensis-longicorpus* or *C. hankugensis-Iksookimia longicorpus*), two diploid parapatrically distributed bisexual species, *C. hankugensis* (HH, 2*n* = 48 chromosomes) and *I. longicorpa* (LL, 2*n* = 50 chromosomes) meet in a hybrid zone and form diploid hybrids (HL, 2*n* = 49 chromosomes) (Fig. 1)^37,38,44^. Despite the differences in chromosomal number^37,38,44^, morphology, and the number of species-specific genetic markers^41,45,46^, the recognition of parental genomes is highly important in the hybrids. Previous crossing experiments, followed by the analysis of ploidy, morphology, and karyotypes of progenies, suggested that diploid hybrid females produced diploid eggs^41,43^. Such diploid eggs incorporate the sperm genome from available sperm donors and form triploid progeny with two types of genome compositions, namely HHL (3*n* = 73 chromosomes) and HLL (3*n* = 74 chromosomes) (Fig. 1)^38,41,43^. The back-crosses of triploid females in *C. hankugensis-I. longicorpa* hybrid complex to males of their parental species, by contrast, resulted in either diploid HH and LL progeny, depending on which paternal species fertilized their eggs (Fig. 1)^37,41,43^. This indicates that triploids may selectively eliminate one parental’s genome during their gametogenesis and form recombined haploid gametes. Hybrid males are rare, about 3%, in natural habitats of hybrid complexes and confirmed to be sterile by histological observations^45^. However, occasional sperm was observed, suggesting that some germ cells can undergo meiosis^46^.

The aforementioned patterns indicate that investigation of *Cobitis hankugensis-Iksookimia longicorpa* hybrid complex may deliver crucial insight into cellular mechanisms driving non-Mendelian reproductive outcomes in natural diploid and triploid hybrids. However, as in many other taxa, much of the knowledge about this system has been gained by crossing experiments and genetic/ploidy analyses of progeny without detailed investigation of meiocytes. To leverage this hybrid complex as a suitable model, we here aimed to develop a new FISH approach with chromosome-specific satDNA markers, thereby enabling precise recognition of parental subgenomes. With the utilization of recently developed tools, we employed immunofluorescent staining and FISH to differentiate the ploidy levels of germ cells in hybrids at crucial stages of both premeiotic development (gonial cells) and meiosis, specifically at pachytene and diplotene stages. This comprehensive analysis allowed us to pinpoint the timing of specific gametogenic alterations leading to the formation of clonal unreduced gametes in diploid hybrids and to selective genome elimination and formation of recombined gametes in triploid females. Of equal importance, our research revealed the underlying mechanisms leading to hybrid sterility in triploid males. Applied methodology has significantly advanced our understanding of mechanisms underlying clonal reproduction in diploid hybrids and genome elimination in triploid hybrids. In addition, it has furnished crucial insights into the mechanisms of triploid male sterility, marking a substantial contribution to our overall knowledge in this field.

## Results

### Sexual species exhibit normal pairing of homologous chromosomes

We found that the somatic cells of *C. hankugensis* have 2*n* = 48 and somatic cells of *I. longicorpa* have 2*n* = 50 chromosomes, consistent with previous findings^37^. In diploid HL hybrids, we observed 2*n* = 49 chromosomes (24 chromosomes from the H genome and 25 chromosomes from the L genome); in triploid HHL hybrids, we detected 3*n* = 73 chromosomes (48 chromosomes from two H genomes and 25 chromosomes from one L genome), consistent with the previous finding^37^. We further employed fluorescence in situ hybridization (FISH) to map earlier developed satellite DNA marker for *Cobitis* species, SatCE1^47^, on chromosomes of *C. hankugensis* and *I. longicorpa*, as well as their diploid and triploid hybrids. Signals were identified in two middle-size acrocentric chromosomes of *I. longicorpa* and in two large metacentric chromosomes of *C. hankugensis* (Supplementary Fig. S1a, b). In triploid HHL hybrids, two signals on metacentric HH chromosomes and one signal on acrocentric L chromosome were clearly distinguished (Supplementary Fig. S1c). Thus, FISH mapping of chromosome-specific SatCE1 tandem repeat marker proved to be a reliable tool for identifying chromosomes of parental species in hybrid karyotypes (Supplementary Fig. S1c).

**Fig. 2:**
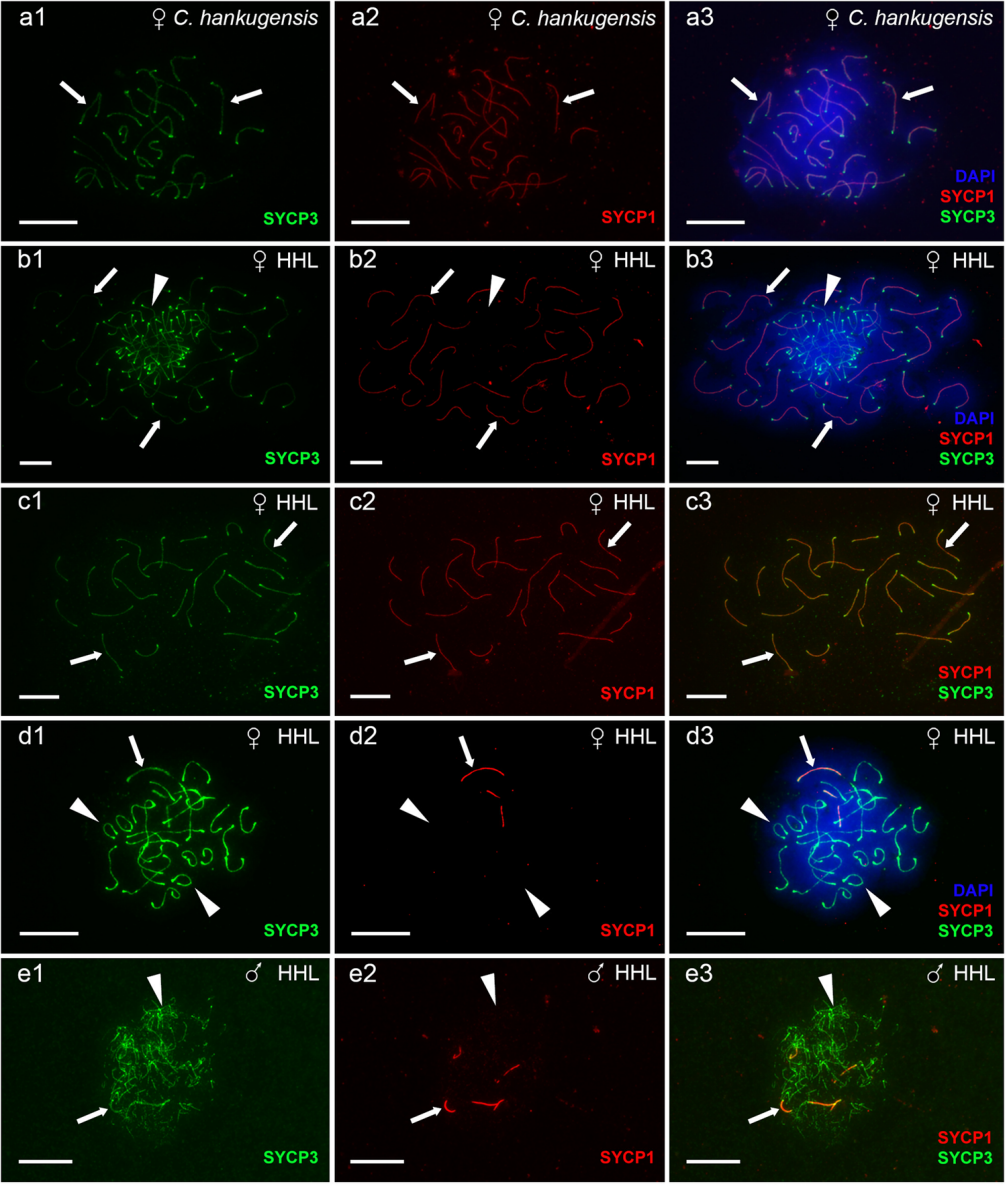
The analysis of pairing in pachytene oocytes (a1–d3) and spermatocytes (e1–e3) of *C. hankugensis* (a1–a3) and triploid HHL hybrids (b1–e3). Synaptonemal complexes were visualized using immunostaining of lateral (SYCP3 protein, green) (**a1**, **b1**, **c1**, **d1**, and **e1**) and central (SYCP1 protein, red) (**a2**, **b2**, **c2**, **d2**, and **e2**) components. Corresponding merged figures (**a3**, **b3**, **c3**, **d3**, and **e3**) also include DAPI staining (blue). Accumulation of SYCP3 and SYCP1 proteins (indicated by thick arrows) allows distinguishing bivalents, while univalents accumulate only SYCP3 protein (indicated by arrowheads). Pachytene oocytes of *C. hankugensis* exhibit 24 fully paired bivalents (**a1**–**a3**). In triploid hybrids, we observed pachytene oocytes with 24 bivalents and 25 univalents (**b1**–**b3**), oocytes with 24 bivalents (**c1**–**c3**), and oocytes with 25 univalents (**d1**–**d3**). Triploid hybrid males exhibit pachytene oocytes only with the aberrant pairing of several bivalents and univalent (**e1**–**e3**). Scale bar = 10 µm.

**Fig. 3:**
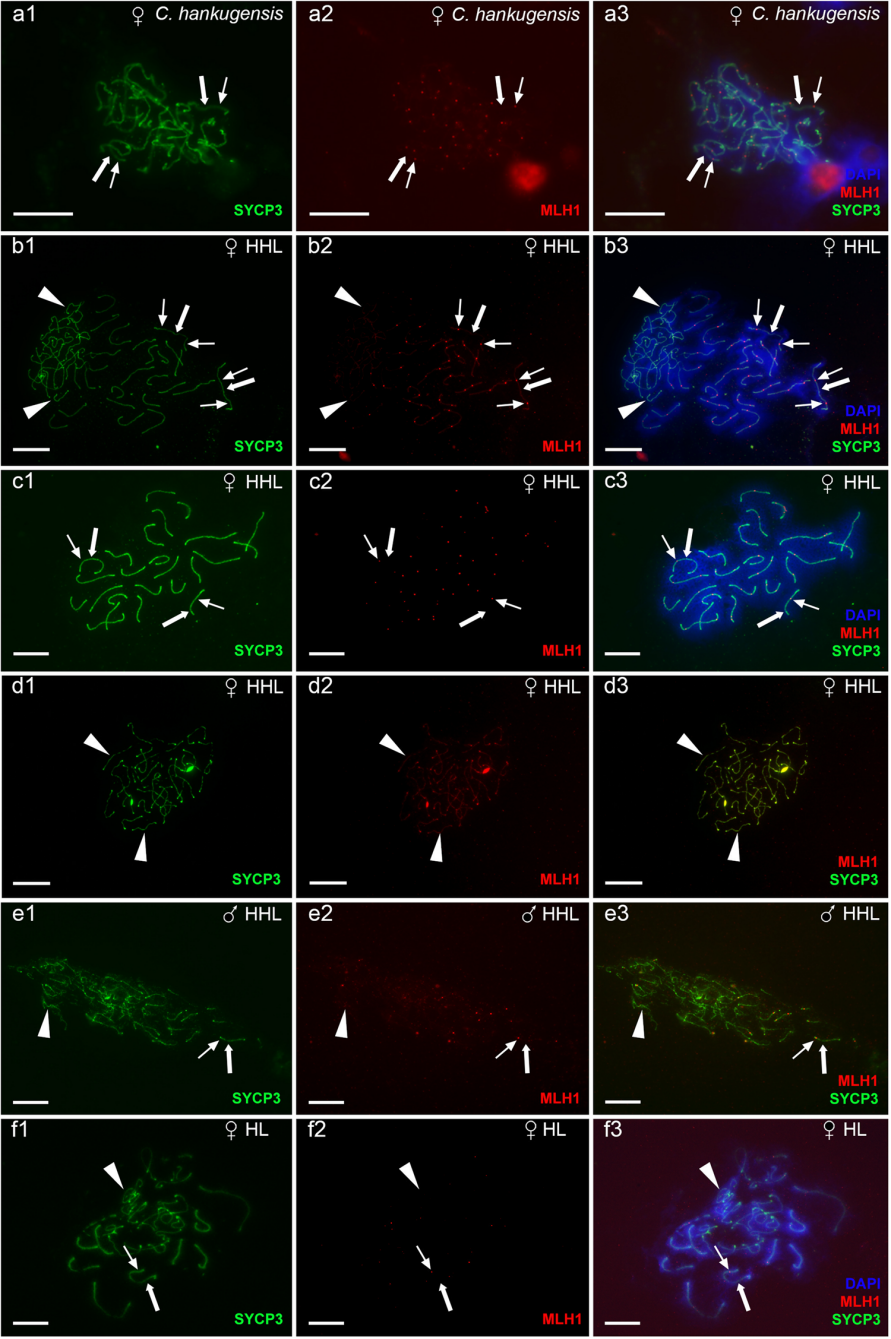
The analysis of crossover loci in pachytene oocytes (a1–d3, f1–f3) and spermatocytes (e1–e3) from gonads of *C. hankugensis* (a1–a3), triploid HHL hybrids (b1–e3) and diploid hybrid (f1–f3). Crossover loci were detected by MLH1 protein (indicated by thin arrows, red) (**a2**, **b2**, **c2**, **d2**, **e2**, and **f2**) on lateral components of synaptonemal complexes (SYCP3 protein, green) (**a1**, **b1**, **c1**, **d1**, **e1**, and **f1**). Corresponding merged figures (**a3**, **b3**, **c3**, **d3**, **e3**, and **f3**) also include DAPI staining (blue). MLH1 bindings (indicated by thin arrows, red) are located on bivalents (indicated by thick arrows) and do not accumulate on univalents (indicated by arrowheads). Pachytene oocytes of *C. hankugensis* exhibit 24 fully with at least one crossover locus per bivalent (**a1**–**a3**). In triploid hybrids, oocytes with 24 bivalents and 25 univalents have MLH1 signals only on bivalents (**b1**–**b3**). Oocytes with exclusively 24 bivalents (**c1**–**c3**) have recombination signals on each bivalent, while oocytes with exclusively 25 univalents (**d1**–**d3**) do not have crossover locus. MLH1 immunostaining demonstrates the presence of crossover in individual bivalents formed in a triploid hybrid male (**e1**–**e3**) and pachytene oocytes with an unduplicated genome (**f1**–**f3**). Scale bar = 10 µm.

We investigated gametogenesis in both sexual species of studied hybrids, including three males and four females of *C. hankugensis* and one male and four females (three adults and one juvenile) of *I. longicorpa* (Supplementary Table S1). During the pachytene chromosome analysis, no differences in the number of bivalent formations were observed between males and females of each parental species. To confirm bivalent formation during the pachytene stage of the sexual species, we stained the axial (SYCP1) and lateral (SYCP3) elements of the synaptonemal complexes. In males and females of both parental species, we observed the same number of chromosomes as in their somatic cells, paired into bivalents with no univalent or aberrant pairing. In males and females of *C. hankugensis*, we detected 24 bivalents, and in males and females of *I. longicorpa*, we observed 25 bivalents (Fig. 2a, Supplementary Fig. S2a–c). We visualized crossing over loci on pachytene spreads of both males and females, with at least one signal per each bivalent. In males of *C. hankugensis* and *I. longicorpa*, MLH1 loci were usually observed at distal parts of bivalents in contrast to females, where interstitial localization of MLH1 loci was more common (Fig. 3a, Supplementary Fig. S3a–b). To confirm the results of pachytene analysis, we analyzed diplotene oocytes in two additional *C. hankugensis* females and two additional *I. longicorpa* females (Supplementary Table S1). We found 24 bivalents in *C. hankugensis* females and 25 bivalents in *I. longicorpa* females with chiasmata corresponding to crossover loci (Supplementary Fig. S4a, b). FISH with SatCE1 marker confirmed the pairing between homologous chromosomes in both parental species. We observed a signal on each chromosome in a particular bivalent in diplotene chromosomal spreads of *C. hankugensis* and *I. longicorpa* (Supplementary Fig. S5a, b).

Furthermore, we investigated gonadal microanatomy by confocal microscopy and revealed the distribution of gonial cells, meiocytes, and gametes in two males and two females of *C. hankugensis* and one male and two females of *I. longicorpa* (Supplementary Table S1, Supplementary Fig. S6a-d). In *C. hankugensis* and *I. longicorpa* females, we observed oogonia and pachytene clusters between previtellogenic and vitellogenic oocytes. In *C. hankugensis* and *I. longicorpa* males, we detected different clusters of spermatogonia, spermatocytes during pachytene and spermatocytes during metaphase I, and large clusters of spermatids. The morphology of their nuclei discriminated different cell types after DAPI staining according to the previously published results for *Cobitis* species^17,24,48^.

We also identified the ploidy of gonial cells, pachytene oocytes, and early diplotene oocytes in sexual species using whole mount FISH with satDNA marker SatCE1 (Supplementary Table S1, Supplementary Fig. S7). In oogonia (*n* = 27) of sexual species, we distinguished two signals suggesting their diploid genome composition (Supplementary Fig. S7c). One large signal in pachytene cells (*n* = 116) indicated bivalent formation between homologous chromosomes (Supplementary Fig. S7b). In small (nucleus diameter 8–15 µm; *n* = 71) and larger (nucleus diameter 15–40 µm; *n* = 93) diplotene oocytes, two adjacent signals were distinguished, indicating the separation of homologous chromosomes in particular bivalent (Supplementary Fig. S7a).

### Diploid hybrid females exploit premeiotic genome endoreplication and produce unreduced eggs

**Fig. 4:**
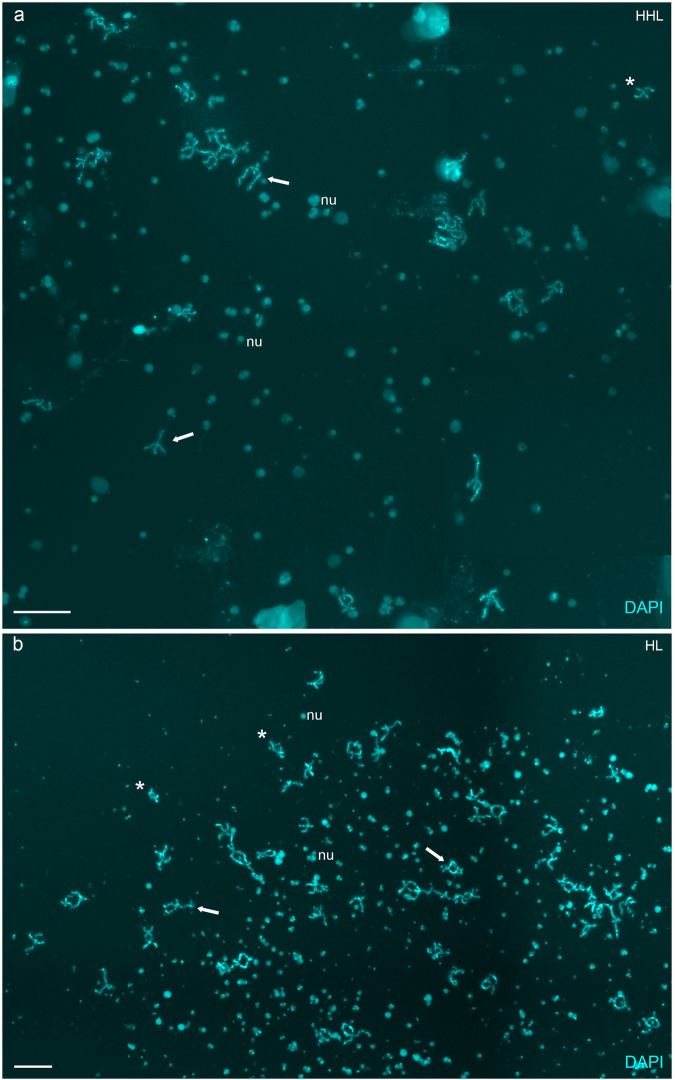
Diplotene chromosomal spreads from the individual oocytes of triploid HHL (a) and diploid HL (b) hybrid females. A triploid hybrid’s chromosomal set of diplotene oocytes includes 24 bivalents, possibly of *C. hankugensis* (**a**). The chromosomal set of diploid hybrid includes 49 bivalents (**b**). Since the chromosomal spread from the individual oocyte was large, four images were merged into one in the case of (**a**) and (**b**). Chromosomes were stained with DAPI (cyan). Thick arrows indicate examples of individual bivalents; nu shows examples of extrachromosomal nucleoli (nu). Asterisks indicate enlarged bivalents in Supplementary Fig. S5c (HHL) and S5d and S5e (HL) for triploid and diploid hybrids, respectively. Scale bar = 50 µm.

To identify the gametogenic pathway in diploid hybrids, we determined the number of bivalents in pachytene and diplotene oocytes of three diploid hybrid females (Supplementary Table S1). After preparation of chromosomal spreads from 36 diplotene oocytes from one diploid hybrid female, we detected exclusively oocytes with 49 bivalents, suggesting their tetraploid state (4*n* = 98 chromosomes) (Fig. 4b). FISH with SatCE1 DNA marker showed signals in both bivalents corresponding to *C. hankugensis* and *I. longicorpa* (Supplementary Fig. S5d, e), suggesting the pairing between chromosomal copies emerged after premeiotic genome duplication.

In contrast to diplotene oocytes, in pachytene oocytes spreads, we observed only diploid oocytes (2*n* = 48 chromosomes) among 13 analyzed oocytes from two hybrid females (Fig. 3f). Diploid pachytene cells exhibited aberrant pairing with 3-5 bivalents, while other chromosomes remained unpaired (Fig. 3f). This suggests that such oocytes possessed unduplicated genomes with 24 chromosomes of *C. hankugensis* and 25 chromosomes of *I. longicorpa*.

**Fig. 5:**
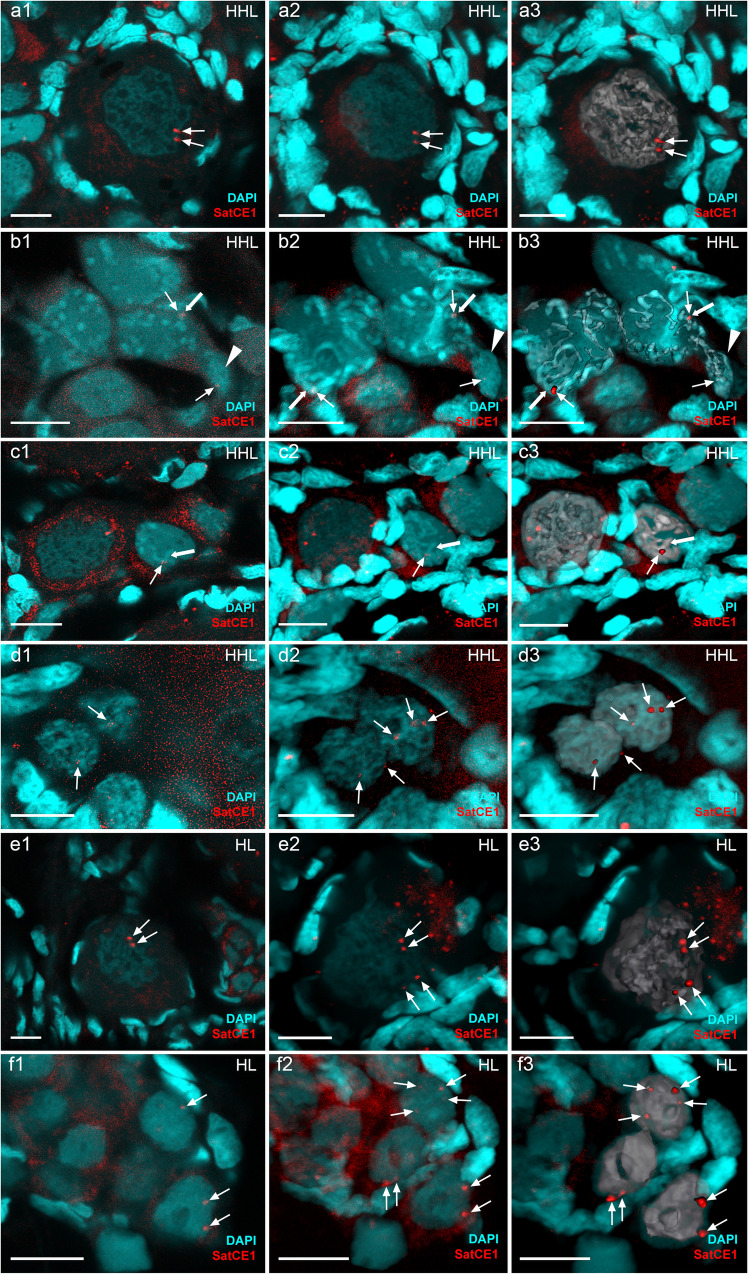
Identification of ploidy level of cells in gonadal fragments of triploid HHL (a1–d3) and diploid hybrids (e1–f3) using whole-mount FISH with chromosome-specific SatCE1 marker. In the diplotene oocyte of triploid HHL hybrid (**a1**–**a3**), two adjacent signals are visible, suggesting the presence of two homologous chromosomes. Pachytene oocytes with bivalents and univalents (**b1**–**b3**) have signals on bivalent (indicated by thick arrow) as well as on univalent (indicated by arrowhead). Pachytene oocytes only with bivalents have one signal (indicated by an arrow) on bivalent (indicated by a thick arrow) (**c1**–**c3**). Diploid oogonia with two signals (indicated by arrows) and triploid oogonia with three signals (indicated by arrows) (**d1**–**d3**) are shown in the ovary from the triploid HHL hybrid. In the diplotene oocyte of diploid HL hybrid (**e1**–**e3**), two pairs of signals are visible, suggesting the presence of two bivalents. Diploid oogonia with two signals (indicated by arrows) and tetraploid oogonia with four signals (indicated by arrows) (**f1**–**f3**) in the ovary from diploid HL hybrid. DNA is stained by DAPI (cyan). Images (**a1**, **b1**, **c1**, **d1**, **e1**, and **f1**) are single confocal sections of 0.7 µm in thickness; corresponding 3D reconstructions (**a2**, **b2**, **c2**, **d2**, **e2**, and **f2**) and 3D surface reconstructions (**a3**, **b3**, **c3**, **d3**, **e3**, and **f3**) of metaphase plates with constructed isosurfaces of the signals and cells of interest. Scale bar = 10 µm.

To identify the ploidy of gonial cells and confirm results obtained by pachytene and diplotene oocyte spreads, we analyzed gonadal microanatomy by confocal microscopy for three diploid hybrid females. We revealed the presence of all cell types similar to those in parental species (Supplementary Table S1, Supplementary Fig. S6g). To test whether genome endoreplication occurs before meiosis in diploid hybrid females and confirm the results obtained from pachytene and diplotene, we applied FISH with SatCE1 DNA marker to identify ploidy level in oogonia, pachytene, and early diplotene oocytes (Fig. 5e, f). Identifying the ploidy level in pachytene and diplotene oocytes using 3D FISH showed identical results to the observations of pachytene and diplotene chromosomal spreads described earlier for all studied diploid hybrid females. During the analysis of oogonia in all diploid HL hybrids, we detected two types of cells: those with two signals (*n* = 346) and those with four signals (*n* = 21). These two different types suggest the presence of diploid and tetraploid oogonia populations, respectively (Fig. 5f). Thus, we demonstrated that tetraploid oogonia emerged after premeiotic genome endoreplication during the gametogenesis of studied diploid hybrid females.

### Triploid hybrid females perform premeiotic genome elimination and produce recombinant haploid eggs

To identify gametogenic pathways in triploid hybrids, we investigated pachytene and diplotene oocytes in 15 triploid hybrid females of both HHL and HLL genome compositions (Supplementary Table S1). After analysis of 77 diplotene oocytes by preparing chromosomal spreads from seven triploid HHL hybrid females, we observed 24 bivalents corresponding to the *C. hankugensis* genome with no univalents or abnormal pairing (Fig. 4a). These results suggest that *I. longicorpa* chromosomes were eliminated before the diplotene stage of meiosis, while two sets of *C. hankugensis* chromosomes formed 24 bivalents. The presence of chiasmata between paired chromosomes confirms the incidence of recombination between putatively homologous chromosomes. FISH with SatCE1 DNA marker showed signals in two chromosomes from one bivalent, suggesting the pairing of homologous chromosomes (Supplementary Fig. S5c).

**Fig. 6:**
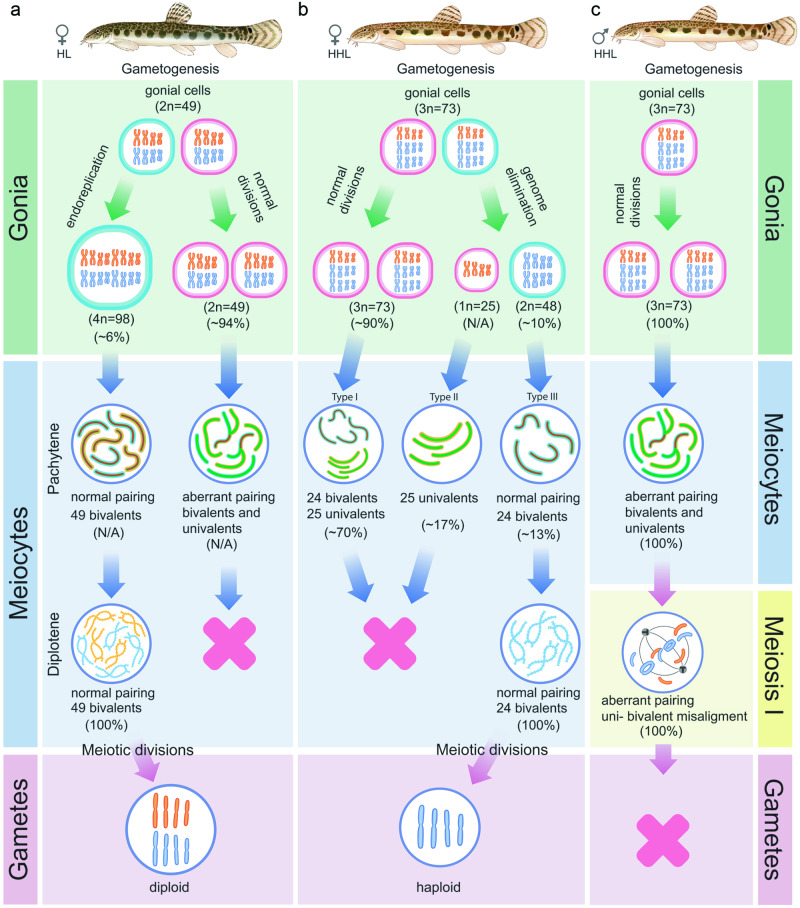
Schematic overview of gametogenesis of diploid and triploid hybrids within *C. hankugensis-I. longicorpa* complex. **a** Diploid hybrid females (HL) have premeiotic genome endoreplication in the minor portion of oogonia. This process enables the formation of bivalents during the pachytene stage of meiosis, as each chromosome has a chromosomal copy to pair with. Afterward, such oocytes progress to diplotene, and upon completing meiosis, they form diploid gametes. **b** In a portion of oogonia from triploid hybrid females (on example of hybrids with HHL genome composition), genome elimination of the ‘L’ genome occurs, leading to the formation of oogonia with HH genome composition (type III) and possibly oogonia with L genome exclusively (type II). Most oogonia retain their original ploidy level without genome elimination (type I). In the pachytene stage of meiosis, type III oocytes have 24 well-paired bivalents; type I oocytes have a mixture of 24 bivalents and 25 univalents; and type II oocytes have univalents with the partial pairing of a few chromosomes. Only type III oocytes proceed beyond pachytene into the diplotene, followed by the formation of reduced haploid gametes. **c** In triploid hybrid males, spermatogonia retain the original ploidy level and do not undergo genome elimination or genome endoreplication. During the pachytene, spermatocytes exhibit aberrant pairings with univalent, bivalent, and multivalent formation. These aberrant spermatocytes advance beyond pachytene and proceed to the first meiotic division. During the metaphase of the first meiotic divisions, individual univalents, bivalents, and multivalents cannot properly attach to the spindle and segregate, causing the spermatocytes to become arrested at this stage of meiosis. Chromosomes of *I. longicorpa* are marked in orange; chromosomes of *C. hankugensis are* marked in blue; green indicates lateral elements in synaptonemal complexes; and red indicates central elements of synaptonemal complexes. The percentage of germ cells and oocytes with identified ploidy levels is presented in brackets; N/A indicates cells for which the percentage was not identified.

We then analyzed 368 pachytene chromosome spreads in 10 HHL triploid hybrid females (Supplementary Table S1). In contrast to diplotene oocytes, the analysis of pachytene oocytes revealed the presence of three distinct cell populations differing in ploidy level (Supplementary Table S1). The first population of cells (*n* = 258) included triploid pachytene oocytes with 24 bivalents formed by *C. hankugensis* chromosomes and 25 univalents representing *I. longicorpa* chromosomes (Fig. 2b; type I in Fig. 6b). FISH with SatCE1 DNA marker clearly distinguished one bivalent between *C. hankugensis* chromosome and one univalent between *I. longicorpa* chromosomes (Supplementary Fig. S8a). We also observed crossing over loci on bivalents but not on univalents (Fig. 3b). The second population of pachytenic oocytes included diploid oocytes with 24 bivalents (*n* = 48) represented by *C. hankugensis* chromosomes (Fig. 2c; type III in Fig. 6b) and exhibited at least one crossing over locus (Fig. 3c). We detected one signal of SatCE1 DNA marker, suggesting the pairing of homologous chromosomes (Supplementary Fig. S8b). Finally, the third population of pachytenic oocytes included haploid oocytes with approximately 25 univalents (*n* = 62), likely represented by *I. longicorpa* chromosomes (Fig. 2d, type II in Fig. 6b). We sometimes observed incomplete pairing between 2 and 3 univalents among these oocytes. Such chromosomal spreads had 0–3 recombination loci only in paired chromosomal parts (Fig. 3d). FISH with SatCE1 DNA probe revealed one signal on a univalent (Supplementary Fig. S8c).

We analyzed gonadal microanatomy by confocal microscopy of six triploid hybrid females (Supplementary Table S1). We observed a similar distribution of oogonia, pachytene, and diplotene oocytes in triploid hybrids and parental species (Supplementary Fig. S6d). Furthermore, we identified ploidy in oogonia and oocytes in intact ovary fragments using whole-mount FISH with SatCE1 DNA marker (Fig. 5a–d; Supplementary Fig. S7d–f). In triploid hybrids with HHL and HLL genotypes, we discriminated oogonia with three signals (*n* = 250) and with two signals (*n* = 29), suggesting the presence of triploid and diploid oogonia populations correspondingly (Supplementary Table S1; Fig. 5d; Supplementary Fig. S7f). The presence of diploid oogonia populations suggests that they emerged by genome elimination before meiosis. We also found triploid (type I) and diploid pachytene oocytes (type II). In triploid pachytene oocytes (type I), we detected one signal on putative bivalent and another signal on putative univalent, which was located among other univalents organized into compact chromatin clump (Fig. 5b, c; Supplementary Fig. S7e). Diploid pachytene oocytes (type II) had one signal only on the bivalent (Fig. 5b, c; Supplementary Fig. S7e). We cannot distinguish between oocytes with 25 univalents and 24 bivalents based only on the FISH approach, as both types of cells have single signals. However, using the morphology of chromosomes in pachytene oocytes, we suggest that oocytes with one signal include bivalents only (type III). In diplotene oocytes (*n* = 465), we observed two adjacent signals similar to diplotene oocytes of parental species, suggesting they are diploid (Fig. 5a; Supplementary Fig. S7d).

In summary, our analysis of triploid hybrid females suggests that genome elimination does not occur between pachytene and diplotene stages but takes place premeiotically. In contrast to the diploid hybrids, we found no oogonia with endoreplicated genomes in triploids. To formally test whether incidences of cells with endoreplicated genomes differ among diploid and triploid hybrids, we compared the counts of duplicated and non-duplicated oogonia using the generalized linear model with binomial error distribution. The differences were highly significant (*p* = 1.65e−05), whereby diploids possess ~6% of duplicated gonial cells, while HHL and HLL triploids had none.

### Triploid hybrid males are sterile due to aberrant pairing of chromosomes

We analyzed gametogenesis in four triploid hybrid males (Supplementary Table S1). The analysis of 24 pachytene spermatocytes from two triploid hybrid males revealed incomplete pairing with 6–13 properly formed bivalents (Fig. 2e). SYCP3 was predominantly localized to subtelomeric regions in some bivalents, while inner chromosome fragments lacked SYCP3 signals (Fig. 2e). The analysis of crossing over loci revealed 4–10 MLH1 loci per bivalent (Fig. 3e). We conclude that the studied hybrid males had aberrant pairing, with only a few chromosomes capable of forming bivalents.

To further investigate the ability of spermatocyte I to undergo meiotic divisions and assess their ability to form spermatids and spermatozoa, we investigated the gonadal microanatomy of four triploids using confocal microscopy. In the gonads of triploid hybrid males, we detected spermatogonia, pachytene cells, and large clusters of cells during metaphase I. No spermatids were observed (Supplementary Fig. S6e). We also found clusters of cells with aberrant chromatin distribution and possibly apoptotic. After spindle visualization, we observed that spermatocytes during MI had misaligned bivalents or univalents.

## Discussion

The present study investigated gametogenesis in diploid and triploid asexual hybrids and demonstrated that switches between asexual and sexual reproduction of hybrids may occur instantly in dependence on their ploidy level. Upon inspecting the genome composition of pachytenic and diplotenic oocytes and gonial cells in natural diploid and triploid asexuals from the *C. hankugensis-I. longicorpa* complex, we demonstrated a dynamic alteration between a clonal reproductive mode in diploids and a recombinant reproductive mode in triploids.

As in many hybrids investigated to date, e.g.^49–51^, a combination of diverged parental genomes caused problems pairing orthologous chromosomes, leading to an arrest in the pachytene of most meiocytes in *C. hankugensis-I. longicorpa* hybrid females (Fig. 6; see below). However, diploid and triploid hybrid females possess specific gametogenic alterations that can restore their fertility. Fertility of diploid *C. hankugensis-I. longicorpa* hybrid females is achieved by genome endoreplication, which occurs in a portion of their oogonia before meiosis and causes the emergence of tetraploid oogonia capable of completing meiosis and forming diploid clonal gametes (Fig. 6a). Premeiotic genome endoreplication is an efficient mechanism to alleviate pairing problems during meiotic prophase and simultaneously gain clonal reproduction^17,19,24^. Moreover, it seems to be a quite universal trait of hybrid asexual vertebrates, observed in natural clonal lineages of loaches^18,19^, and other fish, amphibians, and reptiles^16,20–22,34,52,53^.

Surprisingly, unlike diploid hybrids, triploid hybrids of the same parental species are unable to follow the genome endoreplication pathway, and their fertility relies on different gametogenic alterations (Fig. 6b). We demonstrated, for the first time, that in meiocytes of triploid females with HHL and HLL genome composition, a single-copied genome (*I. longicorpa or C. hankugensis*, respectively) undergoes eliminate before meiosis. Following this, bivalents are formed between double-copied genomes ensuring the progression through meiosis and the formation of reduced recombinant haploid gametes (Fig. 6b). Such gametogenic alteration in triploid hybrids is known as meiotic or triploid hybridogenesis^31^ and has been suggested to occur in several fish^15,31,35–38^ and amphibian^22,52,54,55^ hybrid complexes. However, until now, it has not been thoroughly investigated for most such complexes^56^.

The unraveled mechanism of gametogenesis in triploid hybrids, consistent with predictions from previous crossing experiments^37,38,41,43^ may explain the massive bi-directional introgression of mitochondrial genomes between parental species without nuclear admixture (Fig. 1)^38,57^. Earlier studies of triploid fish hybrids of the genus *Squalius* reported similar results, i.e., pachytene cells with both univalents and bivalents, but hypothesized the genome elimination occurs during meiosis^58^. However, this hypothesis was based on a low number of analyzed pachytenic oocytes (36 oocytes from two individuals) and only one method of their analysis, which may be insufficient to detect the existence of several different populations of oocytes. Meiotic elimination of single copied genome was also suggested in triploid hybrids of *Misgurnus anguillicaudatus* obtained from laboratory crosses between a sexual female and a tetraploid hybrid male^40^. Natural triploids, however, had only genome endoreplication with no evidence of genome elimination^39^. In contrast to these hypothesized mechanisms, our study suggests that several fundamentally different gametogenic pathways may co-occur within a singe individual, at least in triploid Korean loach hybrids. Genome elimination most likely occurs before meiosis, while oocytes without premeiotic genome elimination are arrested during meiosis.

Our study thus emphasizes that diploid and triploid hybrids may employ different gametogenic alterations depending on their ploidy level and genome dosage. A similar switch from gynogenesis in diploid hybrids to triploid hybridogenesis was also suggested for *Squalius* and *Phoxinus* hybrid complexes^31,36,59^. However, the case of spined loaches suggests that such a process is likely controlled by taxon-specific mechanisms since in closely related *Cobitis taenia-elongatoides* hybrid complex, diploid and triploid hybrids maintain the same type of gametogenic alteration, i.e., premeiotic genome endoreplication. Detailed analysis of gametogenic in unrelated organisms is crucial to understanding the mechanisms and processes of modifications during the gametogenesis of hybrids and their relation to ploidy and genomic dosage.

Although both the premeiotic endoreplication and genome elimination play crucial roles in the reproductive performance of diploid and triploid hybrids, respectively, both types of gametogenic alterations are scarce in all studied hybrids (Supplementary Table S1, Fig. 6a, b). Indeed, in diploids, the premeiotic genome endoreplication occurred in only a minor portion of oogonia *C. hankugensis-I. longicorpa* hybrid (~6%, *n* = 21) while most oocytes had unduplicated genomes (*n* = 359) (Fig. 6a). Since vitellogenic and early diplotene oocytes contained exclusively tetraploid genomes, we suggest that oocytes with unduplicated genomes cannot progress beyond pachytene due to aberrant pairing. These findings are in accordance with results from several other asexual hybrid vertebrates^16,24,60,61^. Interestingly, the ratio of duplicated to unduplicated cells in *C. hankugensis-I. longicorpa* hybrid females (~6%) is similar to that observed in other asexual loaches^16,24^.

Pachytene cells of triploid hybrids also contained several populations of oocytes differing in ploidy level, including diploid (after genome elimination), triploid (without genome elimination), and haploid (Fig. 6b). Nonetheless, the ability to eliminate genomes in triploid HHL and HLL hybrids is also limited to a minor population (approximately 13%, *n* = 77) of oogonial cells. Moreover, the presence of diploid oogonia in triploid hybrids suggest that genome elimination occurs before meiosis at least in some oogonia (Fig. 6b). In diplotene, we did not observe aneuploid oocytes and oocytes with univalents, suggesting the inability of oocytes with univalents to pass the pachytene checkpoint. Similarly, in European *Cobitis* hybrids, we found that oocytes with univalents could not proceed beyond pachytene, possibly due to similar stringency of pachytene checkpoints^17,24,48^.

Genome endoreplication appears to be a common mechanism, possibly sharing similar underlying pathways even among unrelated lineages. However, its molecular and cellular basis remains unexplored. It was hypothesized that genome endoreplication might emerge in oogonia responding to signals from apoptotic pachytene oocytes with unduplicated genomes^16^. Nevertheless, our results challenge this hypothesis, at least for *C. hankugensis-I. longicorpa* hybrid complex as both diploid and triploid hybrids of this complex have numerous pachytene oocytes with aberrant pairings that do not progress into diplotene and undergo apoptosis. However, we did not observe any sign of genome endoreplication in triploid hybrid females in around 1500 analyzed cells. Therefore, we lean toward the earlier hypothesis that it is triggered by aberrations in the cell cycle machinery induced by merging differentiated genomes and regulatory networks^24^. Our previous results from asexual diploid and triploid European loaches suggest that premeiotic endoreplication occurs just one or two divisions before entering meiosis as oogonia and pachytene oocytes with duplicated genomes are rare and do not organize in clusters^24^. Similar patterns have been observed in diploid hybrid females among Korean loach hybrid complex (Fig. 5e, f), supporting the general validity of the hypothesis that premeiotic genome endoreplication occurs before entering meiosis in adult fishes.

In contrast, genome elimination in triploid hybrids presumably occurs at earlier stages of gametogenesis since we detected clusters of diploid oogonia, which might be progenitors of the cell that eliminated one of the parental genomes. Premeiotic genome elimination was previously observed in various asexual complexes such as diploid and triploid water frog hybrids^56,62–64^, diploid carp gudgeon hybrids^53^, *Poeciliopsis monacha lucida* hybrids^35^, and in other animals with programmed DNA elimination^11^. In hybrid and non-hybrid organisms, genome elimination occurs either gradually^53,56,62,65–67^ or simultaneously, including all chromosomes at once^35,68,69^. Gradual chromosome elimination is frequently accompanied by micronuclei formation in the cytoplasm of gonial cells^53,56,62,65,67^. However, the cytoplasm of oogonia from adult HHL and HLL hybrid females did not contain micronuclei, suggesting that genome elimination presumably does not occur in adult animals but occurs during juvenile stages. Simultaneous genome elimination was frequently accompanied by the formation of unipolar spindles, ensuring the attachment and segregation of chromosomes from one of the parental species. In contrast, chromosomes from the other parental species usually form a chromatin bulb^35,68,69^. The presence of pachytene oocytes with 25 univalents of *I. longicorpa* (type II) thus allows us to hypothesize that the entire *I. longicorpa* genome is removed simultaneously into separate cells and fails to degrade. The absence of aneuploid oocytes in pachytene and diplotene stages also provides indirect evidence for the simultaneous removal of *I. longicorpa* genome.

Taken together, we suggest that premeiotic genome endoreplication most likely occurs in oogonia one or a few divisions before entering meiosis. In contrast, premeiotic genome elimination may be restricted to early gametogenic stages and does not occur in adult hybrid females. However, a detailed analysis of gonads during different ontogenetic stages is required to elucidate the mechanism of genome elimination in triploid hybrids.

In contrast to hybrid females, triploid hybrid males do not exhibit either genome endoreplication or genome elimination in the spermatogonia (Fig. 6c). In pachytene spermatocytes, we found aberrant pairing with several bivalents, univalents, and multivalents, similar to diploid and triploid male hybrids between European *Cobitis* species and between Japanese species of *Misgurnus* genus^17,51^. Spermatocytes of triploid hybrid HHL males can progress to meiotic metaphase I despite their aberrant chromosome pairing (Fig. 6c). Nevertheless, on gonadal tissue fragments, we observed only rare spermatid and sperm cells, consistent with previous histological observations showing the presence of malformed spermatids of various sizes and a high degree of apoptosis^45^. Interestingly, rare sperm were found in triploid hybrid males, although they exhibited significantly reduced motility compared to parental species^46^. It corresponds to our finding of clusters of spermatocytes in metaphase I with aberrant chromosome attachments to the spindle, possibly due to univalent and multivalent formation during meiosis I. Hence, we hypothesize that only rare spermatocytes can bypass metaphase I, forming aberrant spermatozoa.

Such a prominent sex-specific bias in the ability to trigger asexual gametogenesis may imply the role of genetic sex determination. However, experimental transplantation of spermatogonia from genetically determined hybrid males into females of sexual species within the European loaches hybrid complex restored their ability to endoreplicate genomes in forming gonial cells^70^. This result suggests that the initiation of endoreplication, at least in European *Cobitis*, is not directly linked to the genetic sex determination of the individual but rather to the gonadal environment, being possible only in the ovary. However, in *Misgurnus* loaches, endoreplication occurred in hormonally sex-reverted hybrid males^71^, indicating that genome endoreplication in *Misgurnus* loaches does not depend on the phenotypic sex of the individual but rather is genetically determined. It may suggest that premeiotic endoreplication may proceed differently, even in relatively closely related organisms such as *Cobitis* and *Misgurnus*, making it of utmost importance to gather similar types of data from various cases of hybrid asexuals to disentangle general trends from species-specific mechanisms.

The formation of clonal gametes is a crucial step for asexual reproduction via gynogenesis^48,72,73^. Accumulating number of studies suggest that it may involve instant modifications of gametogenic pathways, such as genome elimination or genome endoreplication, which allows to overcome sterility of hybrid progeny already in the F1 generation^24,48,61,74,75^. Nevertheless, the successful establishment of asexual lineages requires additional alterations of gametogenic and fertilization processes^9,10^. In stable gynogenes, the formation of diploid eggs is usually combined with the capacity to eliminate the sperm genome after fertilization^33,76,77^. Studied *C. hankugensis*-*I. longicorpa* diploid hybrids indeed have clonal gametogenesis and can produce diploid eggs (Figs. 1, 6a). However, they do not form self-maintaining asexual lineages, as their eggs incorporate genetic material from sperm, leading to triploidization of their progeny (Fig. 1)^37,38^. Triploid hybrids also appear unable to achieve clonal reproduction as they produce recombinant gametes after eliminating a single-copied genome (Figs. 1, 6b) (current data^38^). Various gametes can produce new asexual diploid hybrids or sexual diploid individuals with nuclear genomic constitutions of the parental species, depending on the parental species (Figs. 1, 6b)^41,43^. Therefore, while clonal gametogenesis is a necessary step toward asexual reproduction, additional modifications are required to establish self-maintaining asexual lineages.

Although the clonal reproduction of hybrids effectively restricts gene flow between genomes of both parental species^17^, it can facilitate mtDNA exchange between parental species like in the *C. hankugensis-I. longicorpa* complex^38,57,78^. An earlier study indeed reported extensive introgression of the mitochondrial genome of *C. hankugensis* into *I. longicorpa* individuals and vice versa with no evidence of nuclear introgression across the species boundary^57^. Similarly, the transfer of mitochondrial genome was observed in other species exploiting hybridogenetic reproduction^59,78,79^, suggesting a potential advantage of such cyto-nuclear hybrids in expanding the habitats^78^.

In summary, the stable maintenance of *C. hankugensis-I. longicorpa* complex relies on dynamic interactions between hybrids and sexual species and on modifications of gametogenesis which vary between hybrid females with different ploidy levels (Figs. 1, 6a, b). This finding underlines the crucial role that ploidy and genome dosage play in determining the type of gametogenesis executed by hybrids when combining parental genomes. It highlights the importance of understanding these genetic factors and cellular mechanisms, as they can significantly impact the reproductive success of hybrids and ultimately shape the genetic diversity of populations.

## Methods

### Samples studied and preparation of specimens for cytogenetic examination

The hybrid complex of two species of *C. hankugensis* and *I. longicorpa* has been reported in only three restricted localities in Korea^37,47,48,56^. Because the three natural habitats of the hybrid complex are geographically isolated and their origin was also genetically independent^56^, in the present study for cytogenetic examination, we focussed first on one locality of the Ram Stream where pure parental individuals and diploid and triploid hybrids in both sexes are more easily caught than the other two localities. We collected samples of *Cobitis hankugensis* and *Iksookimia longicorpa* and their diploid and triploid hybrids from three sites along the Ram Stream in the province of Unbong-eup and Inwol-myeon Namwon-si Jeollabuk-do in Korea, hoping to involve all the representative types (HH, LL, HL, HHL, and HLL) of the hybrid complex in our field trip in 2019 and 2022 (Supplementary Table S1, Supplementary Fig. S9a, b). This collection effort allowed us to examine 34 fish individuals of various types: 7 HH, 5 LL, 3 HL, 16 HHL, and 3 HLL. Ethical approval for the fish collection and experiments was obtained from the Institutional Animal Care and Use Committee (IACUC) at Ewha Womans University (IACUC permission no. 15-104).

We analyzed gametogenesis in five *I. longicorpa* (one male, three females, one juvenile female) and seven *C. hankugensis* (three males, four females). In addition, we investigated gametogenesis in 19 triploid hybrid individuals (four males, 12 females with HHL, and three females with HLL genotypes) and three diploid HL hybrid females from natural localities. No treatment or injection was used before the investigation of female gametogenesis. Animals were anesthetized in MS222 (Merck), followed by euthanasia according to standard procedures to minimize suffering. Kidneys were used for mitotic metaphase chromosome preparations, while ovaries and testes from each individual were separated into several pieces and used for pachytene or diplotene (in the case of females only) chromosome preparation and whole-mount analysis. For whole-mount analysis, gonadal tissue fragments were fixed in 2% paraformaldehyde in 1× PBS for 90 min at room temperature (RT), washed in 1× PBS, and transferred to 96% methanol for long-term storage.

### Species and ploidy identification

We initially assessed the genomic composition and ploidy type of each specimen through morphological examination of external characteristics and red blood cell size measurements^41,45,46^. Subsequently, we used PCR-sequencing of a species-diagnostic nuclear gene, ectodermal-neural cortex I (*enc 1*), and mitochondrial cytochrome *b* gene (cyt *b*) according to previous genetic studies involving the two species^57,80^. The chromatogram of DNA sequences of the *enc 1* gene could let us know whether the gene sequences are the mixture of the chromosomes of *C. hankugensis* (hereafter ‘H’ type) and *I. longicorpa* (hereafter ‘L’ type). Furthermore, the chromatograms of gene sequences, *enc 1*, could discriminate diploid (HL) from triploid (HHL) hybrids based on the different heights of heterozygous peaks at their variable nucleotide sites. Two types of triploids (HHL and HLL) could also be discriminated by the same principle at their heterozygous nucleotide sites. HHL hybrids showed a general pattern that peak heights of the nucleotides of *C. hankugensis* are higher than those of *I. longicorpa* at heterozygous sites and vice versa in HLL.

### Mitotic and meiotic metaphase chromosome preparation

Metaphase chromosomes were prepared from fish kidneys, according to^47^. Live fish were injected with 0.1% colchicine solution (1 ml/100 g of body weight) 45 min before being sacrificed using an overdose of 2-phenoxyethanol anesthetics agent (Sigma). The kidneys were removed and dissected in 0.075 M KCl at room temperature. The cell suspension was hypotonized for 8 min in 0.075 M KCl and therefore fixed with fixative (methanol: acetic acid 3:1, v/v) and centrifuged at 1200 RPM at room temperature for 10 min. Cells were washed twice in fixative, centrifuged at 1200 RPM, and finally spread onto slides, stained with 5% Giemsa solution for 10 min at room temperature (RT), and checked under the microscope to confirm the number and morphology of chromosomes.

### Pachytene chromosomes and immunofluorescent staining

Spreads of synaptonemal complexes (SC) during the pachytene stage of meiosis were prepared using protocols described by^81^ and^82^. After manual homogenization of female gonads, 20 µl of cell suspension was dropped on SuperFrost® slides (Menzel Gläser). Then 40 µl of 0.2 M sucrose and 40 µl of 0.2% Triton X100 were added for 7 min. Further, cells were fixed for 16 min by adding 400 µl of 2% paraformaldehyde (PFA) and placed vertically to remove the liquid excess. In the case of males, after testes homogenization, 1 µl of cell suspension was placed into a drop (30 µl) of hypotonic solution (1/3 of 1× PBS) preliminary dropped on SuperFrost® slides (Menzel Gläser) for 20 minutes. Afterward, cells were placed vertically in 2% PFA for 4 min. Subsequently, slides with male and female SCs were rinsed in 1× PBS slides for 5 min and stored in 1× PBS until immunofluorescent staining of synaptonemal complexes was conducted.

Lateral components of SCs were detected by rabbit polyclonal antibodies against SYCP3 protein (concentration 1:200, ab15093, Abcam), while the central component of SCs was detected by chicken polyclonal SYCP1 (concentration 1:400, gift from Prof. Sean Burgess;^83^). Using a combination of SYCP3 and SYCP1 antibodies, it is possible to distinguish bivalents from univalents, as SYCP3 is localized on both bivalents and univalents while SYCP1 is accumulated only on bivalents^17,83^. Recombination loci were detected by antibodies against the MLH1 (concentration 1:50, ab14206, Abcam) proteins. Fresh slides were incubated with 1% blocking reagent (Roche) in 1× PBS and 0.01% Tween-20 (ICN Biomedical Inc.) for 20 min, followed by adding primary antibody for 1 h at RT. Slides were washed three times in 1× PBS at RT and incubated in a combination of secondary antibodies: Alexa-594-conjugated goat anti-rabbit IgG (H + L) (concentration 1:200, A-11012, Thermo Fisher Scientific) and Alexa-488-conjugated goat anti-mouse IgG (H + L) (concentration 1:200, A-11001, Thermo Fisher Scientific) diluted in 1% blocking reagent (Roche) on 1× PBS for 1 h at RT. Slides were washed in 1× PBS and mounted in Vectashield/DAPI (1.5 mg/ml) (Vector, Burlingame, Calif., USA).

### Diplotene chromosomes

Diplotene chromosomal spreads (also known as “lampbrush chromosomes”) were isolated from females of parental species as well as diploid and triploid hybrids according to an earlier published protocol^17,24^. After dissection, ovaries from unstimulated females were submerged in the OR2 saline (82.5 mM NaCl, 2.5 mM KCl, 1 mM MgCl2, 1 mM CaCl2,1 mM Na2HPO4, 5 mM HEPES (4-(2-hydroxyethyl)-1-piperazineethanesulfonic acid); pH 7.4). Oocyte nuclei were isolated manually using jeweler forceps (Dumont) in the isolation medium “5:1” (83 mM KCl, 17 mM NaCl, 6.5 mM Na_2_HPO_4_, 3.5 mM KH2PO4, 1 mM MgCl_2_, 1 mM DTT (dithiothreitol); pH 7.0–7.2). Subsequently, the oocyte nuclei were transferred to the “1:4” medium, a one-fourth strength “5:1” medium supplemented with 0.1% PFA and 0.01% 1 M MgCl_2_. In this medium, the nucleus membrane was removed, releasing nucleoplasm into the solution. Nucleoplasm from each oocyte was transferred into glass chambers attached to a slide filled in a “1:4” saline medium. This method ensures that each chamber contains chromosomal spread from the individual oocyte. The slide was then centrifuged for 20 min at +4°C, 4000 rpm in a centrifuge equipped with Swing Bucket Rotor for slides, fixed for 30 min in 2% PFA in 1× PBS, and post-fixed in 50% ethanol for 5 min and 70% ethanol overnight (at +4°C). Afterward, slides were dehydrated in 96% ethanol, air dried, and either used for FISH or mounted in Vectashield/DAPI (1.5 mg/ml) (Vector, Burlingame, Calif., USA) used for direct lampbrush chromosomes observations.

### Fluorescence in situ hybridization and whole mount fluorescence in situ hybridization

For fluorescence in situ hybridization (FISH) procedures, we applied an earlier developed probe to satDNA repeats (satCE1), specific to *C. elongatoides*^47^. Probes were labeled with biotin and digoxigenin by PCR using *C. hankugensis* and *I. longicorpa* DNA isolated from muscle tissue using the Dneasy Blood &Tissue Kit (Qiagen) according to the manufacturer’s protocol.

The hybridization mixture (50% formamide, 10% dextran sulfate, 2× ЅЅС, 5 ng/μl labeled probe, and 10–50-fold excess of salmon sperm DNA) was applied to slides, covered with cover slides, and carefully sealed at the edges with rubber cement. To denature the probe and chromosomal DNA on the slides, we subjected them to a common denaturation step at 75 °C for five minutes and incubated slides overnight at room temperature (RT) in a humid chamber. After hybridization, slides underwent three times in 0.2× SSC at +44 °C for 5 min each. Biotin-dUTP and digoxigenin-dUTP were detected using streptavidin-Alexa 488 (concentration 1:200, S32354, Thermo Fisher Scientific) and anti-digoxigenin-rhodamine (concentration 2 μg/ml, # 11207750910, Merck), respectively. Chromosomal DNA was counterstained with Vectashield/DAPI (1.5 mg/ml) (Vector, Burlingame, Calif., USA).

Whole-mount FISH was performed according to^24^. After storing gonadal fragments in 96% methanol, we washed them three times in 1× PBS for 15 min each. Afterward, tissues were impregnated with 50% formamide, 10% dextran sulfate, and 2× SSC for 3–4 h at 37 °C. After this, tissues were placed in a hybridization mixture consisting of 50% formamide, 2× SSC, 10% dextran sulfate, 20 ng/µl probe, and 10 to 50-fold excess of salmon sperm DNA. Gonadal tissues were denatured at 82 °C for 15 min and incubated for 24 h at RT. Following the hybridization, tissues were washed in three changes of 0.2× SSC at 44 °C for 15 minutes each and blocked-in 4× SSC containing 1% blocking reagent (Roche) in 4× SSC for 1 hour at RT. Biotin-dUTP and digoxigenin-dUTP were detected using streptavidin-Alexa 488 concentration 1:200, S32354, Thermo Fisher Scientific) and anti-digoxigenin-rhodamine (concentration 2 μg/ml, # 11207750910, Merck) correspondingly. The tissues were stained with DAPI (1 mg/ml) (Sigma) diluted in 1× PBS at RT overnight.

### Whole-mount immunofluorescence staining

Whole-mount immunofluorescent staining was performed according to the previously published protocol^24^. Prior to immunofluorescent staining, gonadal fragments were permeabilized by immersing them in a 0.5% Triton X100 in 1× PBS for 4–5 h at RT, followed by rinsed in 1× PBS at RT. After incubation in a blocking solution (1% blocking solution (Roche) dissolved in 1× PBS) for 1–2 h, tissues were transferred into a new blocking solution with the addition of primary antibodies. We used mouse monoclonal antibodies against alfa‐tubulin (concentration 1:100, ab7291; Abcam). Tissues were incubated with primary antibodies overnight at RT. Goat anti-mouse antibodies conjugated with Alexa-488 fluorochrome (concentration 1:200, # A-11001, Thermo Fisher Scientific) were applied for 12 hours at RT. Primary and secondary antibodies were washed in 1× PBS with 0.01% Tween (ICN Biomedical Inc.) for 5 min with shaking. Tissues were stained with DAPI (1 µg/µl) (Sigma) overnight in 1× PBS at RT.

### Wide-field, fluorescence, confocal laser scanning microscopy, and image processing

Whole-mount immunofluorescent staining and whole-mount FISH, intact gonadal fragments were subjected to analysis using confocal laser scanning microscopy to identify different germ cell lines (gonial cells, meiocytes) and their ploidy. In addition, gonadal tissue fragments, stained only with DAPI, were examined by confocal microscopy to observe gonadal microanatomy and the distribution of different germ cell lines throughout the gonad. Tissue fragments were placed in a drop of DABCO antifade solution containing 1 mg/ml DAPI. Confocal laser scanning microscopy was carried out using a Leica TCS SP5 microscope based on the inverted microscope Leica DMI 6000 CS (Leica Microsystems, Germany). Specimens were analyzed using HC PL APO 40 × objective. Diode and argon lasers were used to excite the fluorescent dye DAPI and Alexa488 fluorochrome, respectively. The images were captured and processed using LAS AF software (Leica Microsystems, Germany).

Initially, the 3D FISH signals were manually assessed as the brightest dots within the cell nucleus using Leica Las AF software. Further discrimination of signals within the nucleus was performed by analyzing the 3D volume of cell nuclei created by Imaris 7.7.1 (Bitplane) software. For 3D-volume rendering and surface reconstruction of confocal image stacks, a region of interest (ROI) was cropped, maintaining image voxel dimensions. Isosurfaces of multichannel images were created for each channel separately, with automated parameters threshold for channel intensity cutoffs. ROI isosurfaces were split into separate surface objects for individual nuclei (DAPI channel) or FISH signals (Alexa 488 or rhodamine channels). The results from the counting of isosurfaces for FISH signals (Alexa 488 or rhodamine channels) were compared with the results from the initial manual counting of signals, and in the case of their correspondence, such cells were used for the analysis. In instances where adjacent nuclei were inseparable (DAPI channel), isosurface reconstruction was done via a “manual creation” tab. To highlight germ cells, only surface objects belonging to individual germ cells were retained in the reconstruction.

Chromosomal slides with pachytene and diplotene chromosomes were analyzed by wide-field and fluorescence microscopy. Meiotic chromosomes after FISH and immunofluorescent staining were analyzed using Provis AX70 Olympus microscopes equipped with standard fluorescence filter sets. Microphotographs of chromosomes were captured by CCD camera (DP30W Olympus) with the assistance of Olympus Acquisition Software. Microphotographs were finally adjusted and arranged in Adobe Photoshop CS6 software; Adobe Illustrator was used for scheme drawing.

### Reporting summary

Further information on research design is available in the Nature Portfolio Reporting Summary linked to this article.

### Supplementary information

Supplementary Figs. and Table

Reporting summary

## Data Availability

The authors state that all data necessary for confirming the conclusions presented in the article are represented fully within the article and its Supplementary materials.
