# Supplementary material for: A cyclical switch of gametogenic pathways in hybrids depends on the ploidy level
Source: Commun Biol. 2024 Apr 8;7:424. doi: 10.1038/s42003-024-05948-6 (PMC11001910; doi:10.1038/s42003-024-05948-6)
Supplement: Supplementary file 1 — Supplementary Figs. and Table [file 42003_2024_5948_MOESM1_ESM.pdf]

Supplementary materials

A cyclical switch of gametogenic pathways in hybrids depends on the ploidy level

Dmitrij Dedukh[1], Anatolie Marta[1], Ra-Yeon Myung[2], Myeong-Hun Ko[3], Da-Song Choi[2], Yong-Jin Won[2], Karel Janko[1,4]

Supplementary figures

Figure S1. FISH-based mapping of SatCE1 marker on chromosomes of *I. longicorpa* (a), *C. hankugensis* (b) and triploid HHL hybrid (c). Two chromosomes were visualized in *I. longicorpa* (a), and *C. hankugensis* (b), while in the HHL hybrid, we detected three chromosomes (c), suggesting that SatCE1 marker is chromosome-specific. Arrows indicate FISH signals on two acrocentric chromosomes of *I. longicorpa* (a), on two large metacentric chromosomes of *C. hankugensis* (b) as well as two signals on metacentric HH chromosomes and one signal on acrocentric L chromosome in triploid HHL hybrids (c). Scale bar = 10 µm.

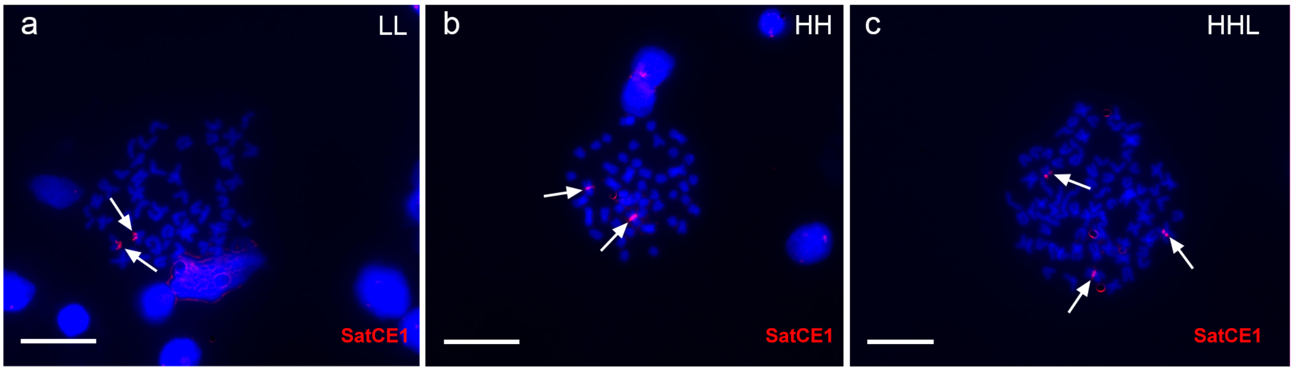
a
LL
SatCE1
b
HH
SatCE1
c
HHL
SatCE1

Figure S2. Synaptonemal complexes formation in *I. longicorpa* (a1-b3) and *C. hankugensis* (c1-c3). Lateral (SYCP3 protein, green) (a1, b1, and c1) and central (SYCP1 protein, red) (a2, b2, and c2) components of synaptonemal complexes were visualized by immunostaining. Corresponding merged figures presented in (a3, b3, and c3) also include DAPI staining (blue). In *I. longicorpa*, 25 bivalents were formed in pachytene oocytes (a1–a3) and spermatocytes (b1–b3). Pachytene spermatocytes of *C. hankugensis* exhibit 24 fully paired bivalents (c1–c3). Arrows indicate bivalents. Scale bar = 10 µm.

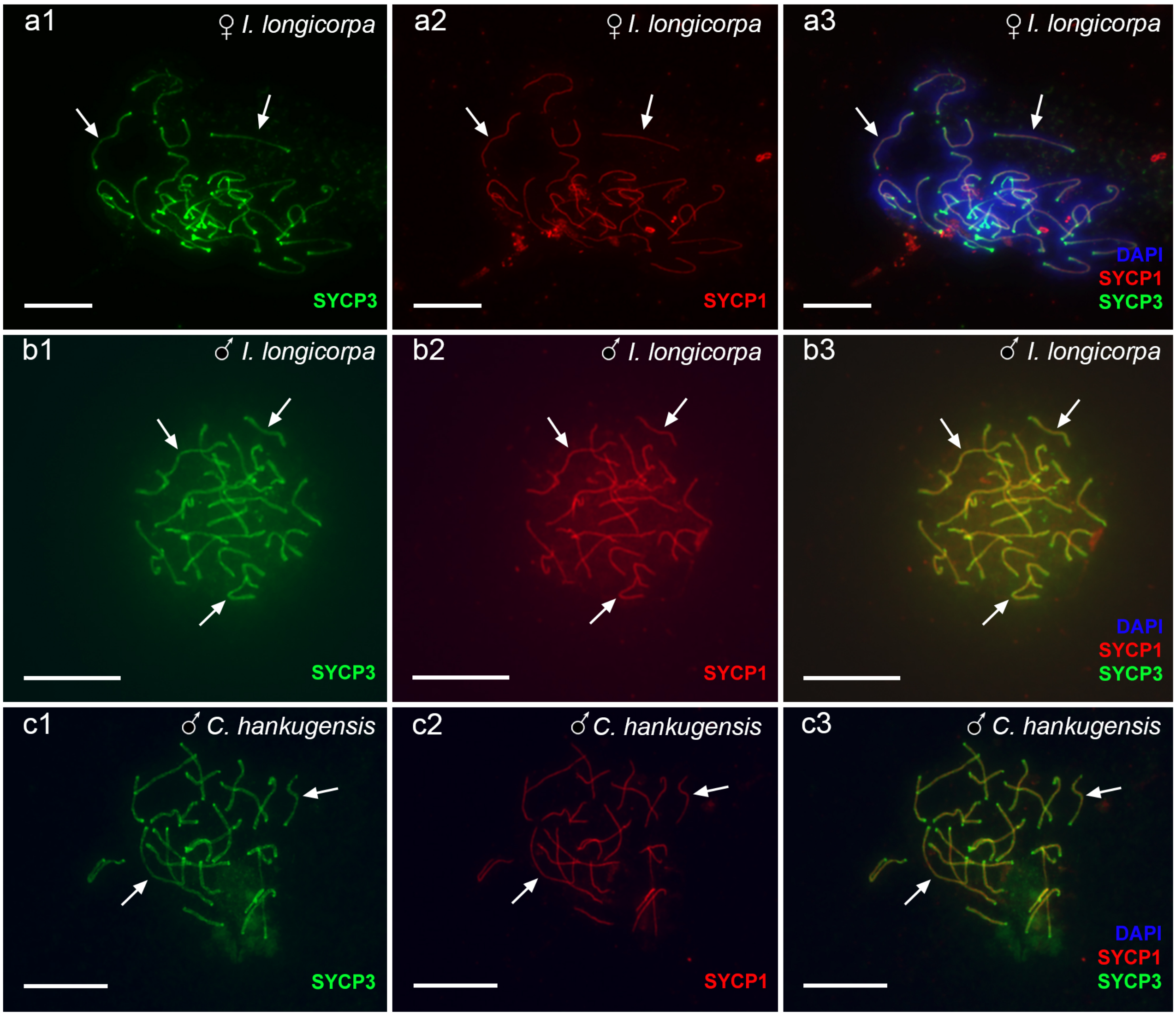
a1
♀ I. longicorpa
SYCP3
a2
♀ I. longicorpa
SYCP1
a3
♀ I. longicorpa
DAPI
SYCP1
SYCP3
b1
♂ I. longicorpa
SYCP3
b2
♂ I. longicorpa
SYCP1
b3
♂ I. longicorpa
DAPI
SYCP1
SYCP3
c1
♂ C. hankugensis
SYCP3
c2
♂ C. hankugensis
SYCP1
c3
♂ C. hankugensis
DAPI
SYCP1
SYCP3

Figure S3. Detection of crossover loci during pachytene in *I. longicorpa* female (a1-a3) and *C. hankugensis* male (b1-b3). Crossover loci were detected by immunostaining of antibodies against MLH1 protein (indicated by arrows, red) (a2, b2) on lateral components of synaptonemal complexes (SYCP3 protein, green) (a1, b1). Corresponding merged figures presented in (a3, b3) also include DAPI staining (blue). All 25 bivalents of *I. longicorpa* and 24 bivalents of *C. hankugensis* have at least one crossover locus per bivalent (A1–B3). Scale bar = 10 μm.

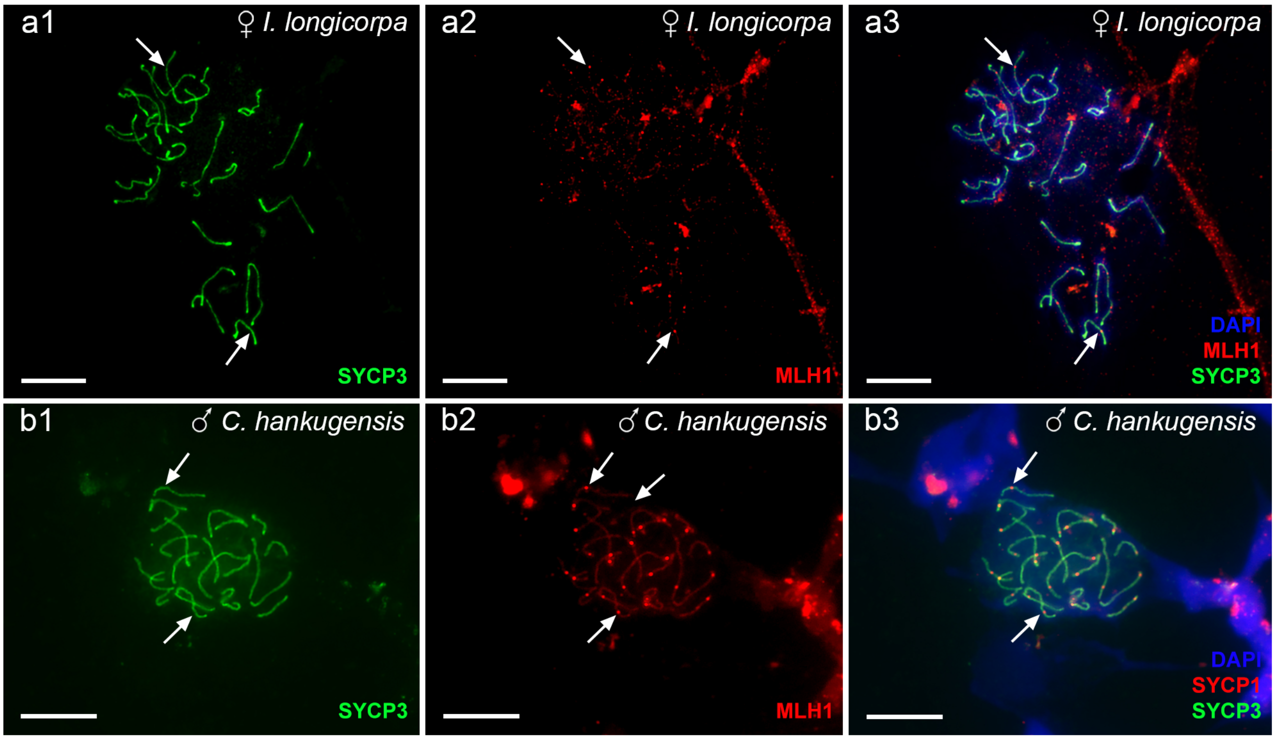
a1
♀ I. longicorpa
SYCP3
a2
♀ I. longicorpa
MLH1
a3
♀ I. longicorpa
DAPI
MLH1
SYCP3
b1
♂ C. hankugensis
SYCP3
b2
♂ C. hankugensis
MLH1
b3
♂ C. hankugensis
DAPI
SYCP1
SYCP3

Figure S4. Diplotene chromosomal spreads from the individual oocytes of *C. hankugensis* (a) and *I. longicorpa* (b) females. Chromosomal sets from diplotene oocytes of *C. hankugensis* (a) and *I. longicorpa* (b) include 24 and 25 bivalents, respectively. Since the chromosomal spread from the individual oocyte was large, four images were merged into one in the case of (a) and (b). Chromosomes stained with DAPI (cyan). Thick arrows indicate examples of individual bivalents; nu shows examples of extrachromosomal nucleoli. Asterisks indicate enlarged bivalents represented in Supplementary Figure S5a and S5b for *C. hankugensis* and *I. longicorpa*, respectively. Scale bar = 50 μm.

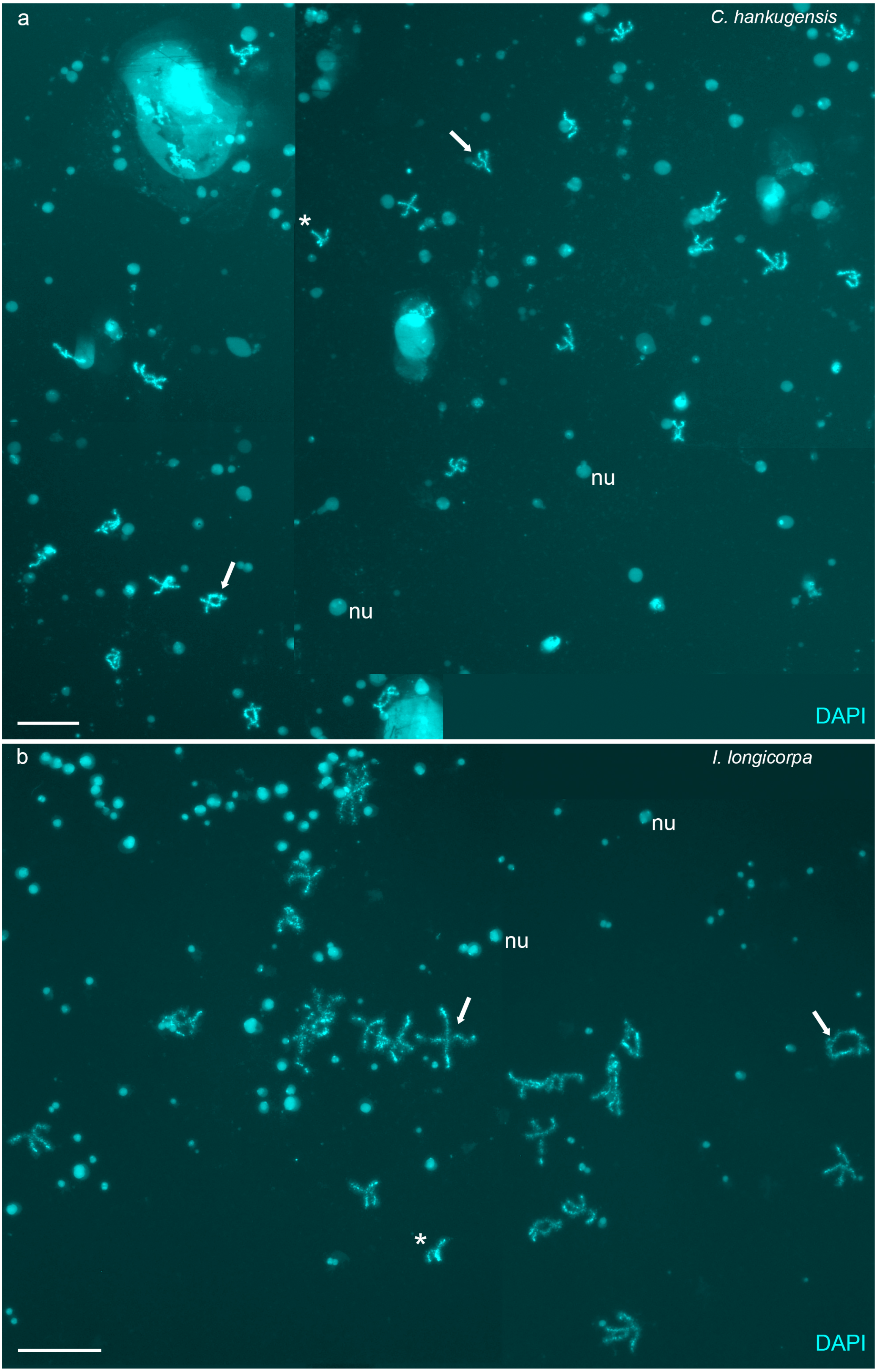
a
C. hankugensis
*
nu
nu
DAPI
b
I. longicorpa
nu
nu
*
DAPI

Figure S5. High-resolution mapping of chromosomes specific marker SatCE1 on individual bivalents of lampbrush chromosomes. Chromosome-specific marker shown in red (indicated by arrows), DAPI stained blue. (a) bivalent of *C. hankugensis* from the full chromosomal set represented in Supplementary Figure S4A. (b) bivalent of *I. longicorpa* from the full chromosomal set represented in Supplementary Figure S4b. (c) bivalent of *C. hankugensis* represented from the full chromosomal set of triploid hybrid represented in Figure 4A. (d) and (e) bivalents of both parental species from the full chromosomal set of diploid hybrid represented in Figure 4b. Scale bar = 10 µm.

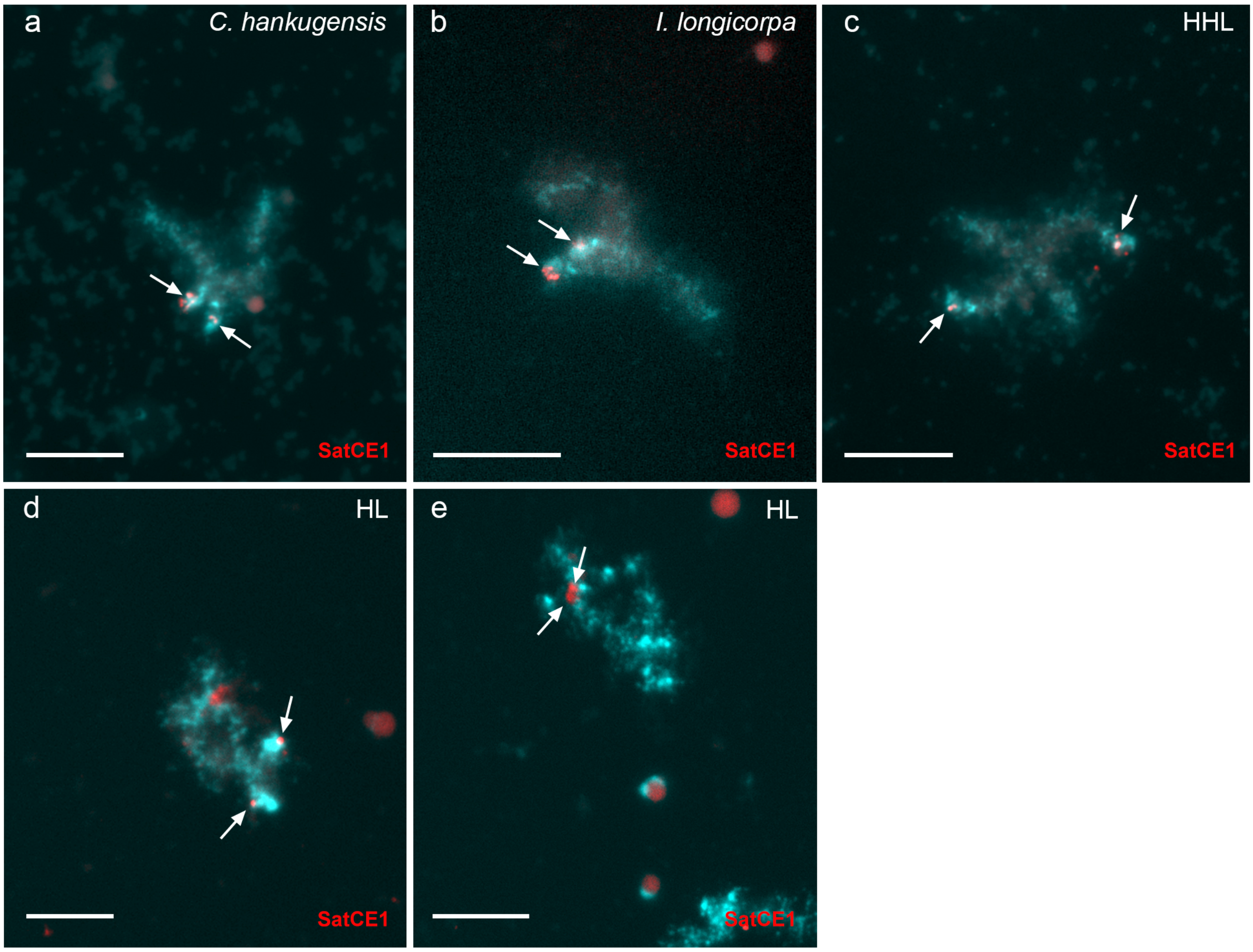
a
C. hankugensis
SatCE1
b
I. longicorpa
SatCE1
c
HHL
SatCE1
d
HL
SatCE1
e
HL
SatCE1

Figure S6. Gonadal microanatomy in *C. hankugensis* (a, b), *I. longicorpa* (c, d), triploid hybrids (e, f) and diploid hybrid female (g). Single confocal sections (a–g) of gonadal fragments and whole-mount immunofluorescent staining (e) with antibodies against tubulin (red). DAPI visualizes chromatin (cyan). Microanatomy of gonads from males (a, c, and e) and females (b, d, f, and g). Several cell types can be determined in the gonadal sections based on the morphology of males (a, c, and e): S – spermatids, P – cells in the pachytene stage of meiotic division, G – gonial cells, M – meiocytes in meiotic division I. Cell types identified in the gonadal sections based on the morphology of females (b, d, f, and g): P – cells in the pachytene stage of meiotic division, G – gonial cells, D – diplotene cells of meiotic division. Scale bar = 50 μm.

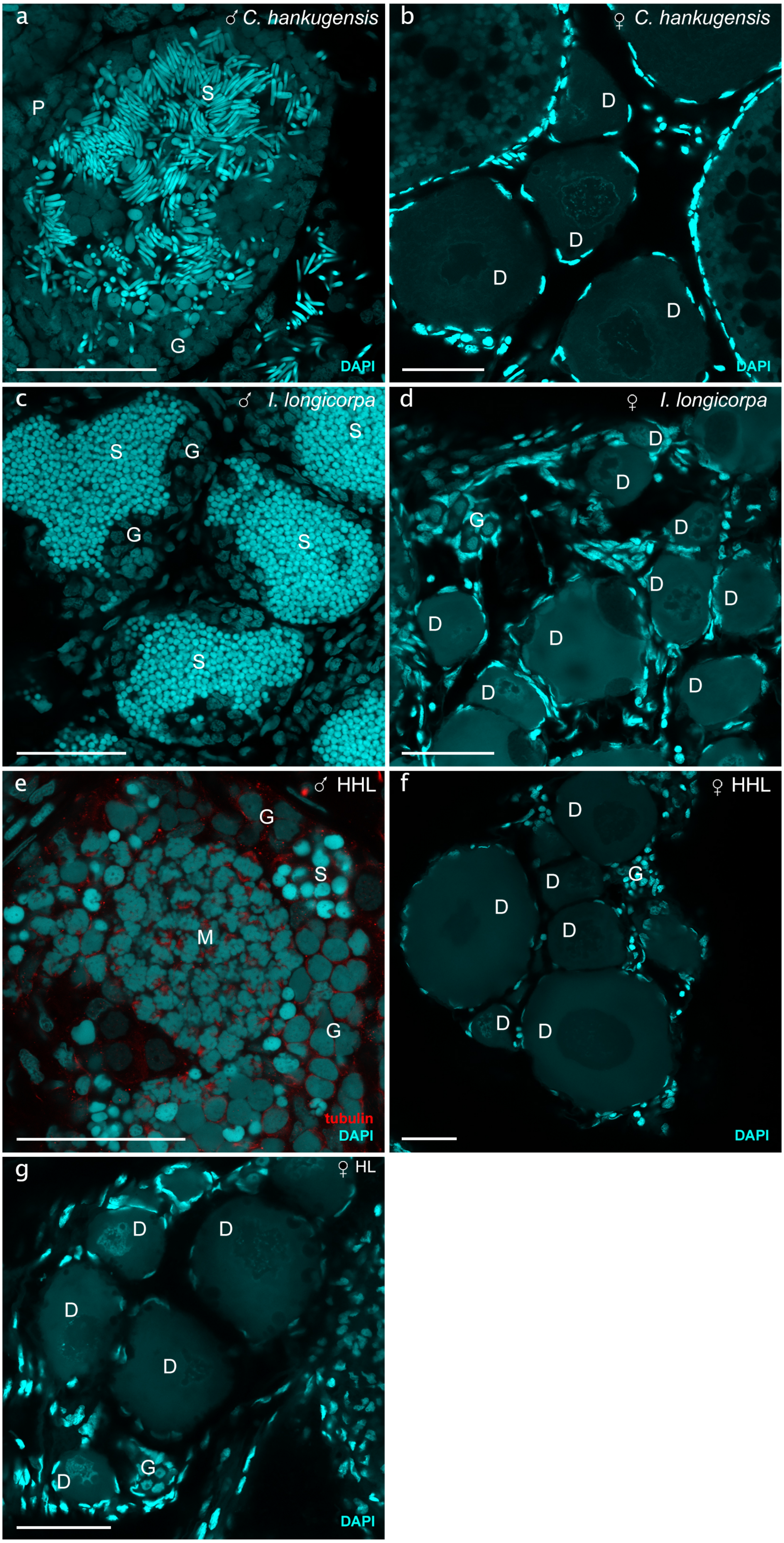
a
♂ C. hankugensis
P
S
G
DAPI
b
♀ C. hankugensis
D
DAPI
c
♂ I. longicorpa
S
G
d
♀ I. longicorpa
D
G
e
♂ HHL
G
S
M
tubulin
DAPI
f
♀ HHL
D
G
DAPI
g
♀ HL
D
G
DAPI

Figure S7. Identification of ploidy level of cells in gonadal fragments of *C. hankugensis* and triploid HLL hybrids using whole-mount FISH with chromosome-specific SatCE1 marker. In the diplotene oocyte of *C. hankugensis* (a1–a3), two adjacent signals are visible, suggesting two homologous chromosomes. Pachytene oocytes (b1–b3) have two adjacent signals (indicated by arrow) on individual bivalent. Diploid oogonia exhibit two signals (indicated by arrows). DNA is stained with DAPI (cyan). Images (a1, b1, and c1) are single confocal sections of 0.7 μm in thickness; corresponding 3D reconstructions (a2, b2, and c2) and 3D surface reconstructions (a3, b3, and c3) of metaphase plates with constructed isosurfaces of the signals and cells of interest. In the diplotene oocyte of triploid HLL hybrid (d1–d3), two adjacent signals are visible suggesting the presence of two homologous chromosomes. Pachytene oocytes with bivalents and univalents (e1–e3) have signals on bivalent (indicated by thick arrow) as well as on univalent, which are localized in a dense cluster (indicated by arrowhead). Pachytene oocytes only with bivalents (upper cell) have one signal (indicated by arrow) on bivalent (indicated by thick arrow) (e1–e3). Diploid oogonia with two signals (indicated by arrows) and triploid oogonia with three signals (indicated by arrows) (f1–f3) in the ovary from triploid HLL hybrid. Scale bar = 10 μm.

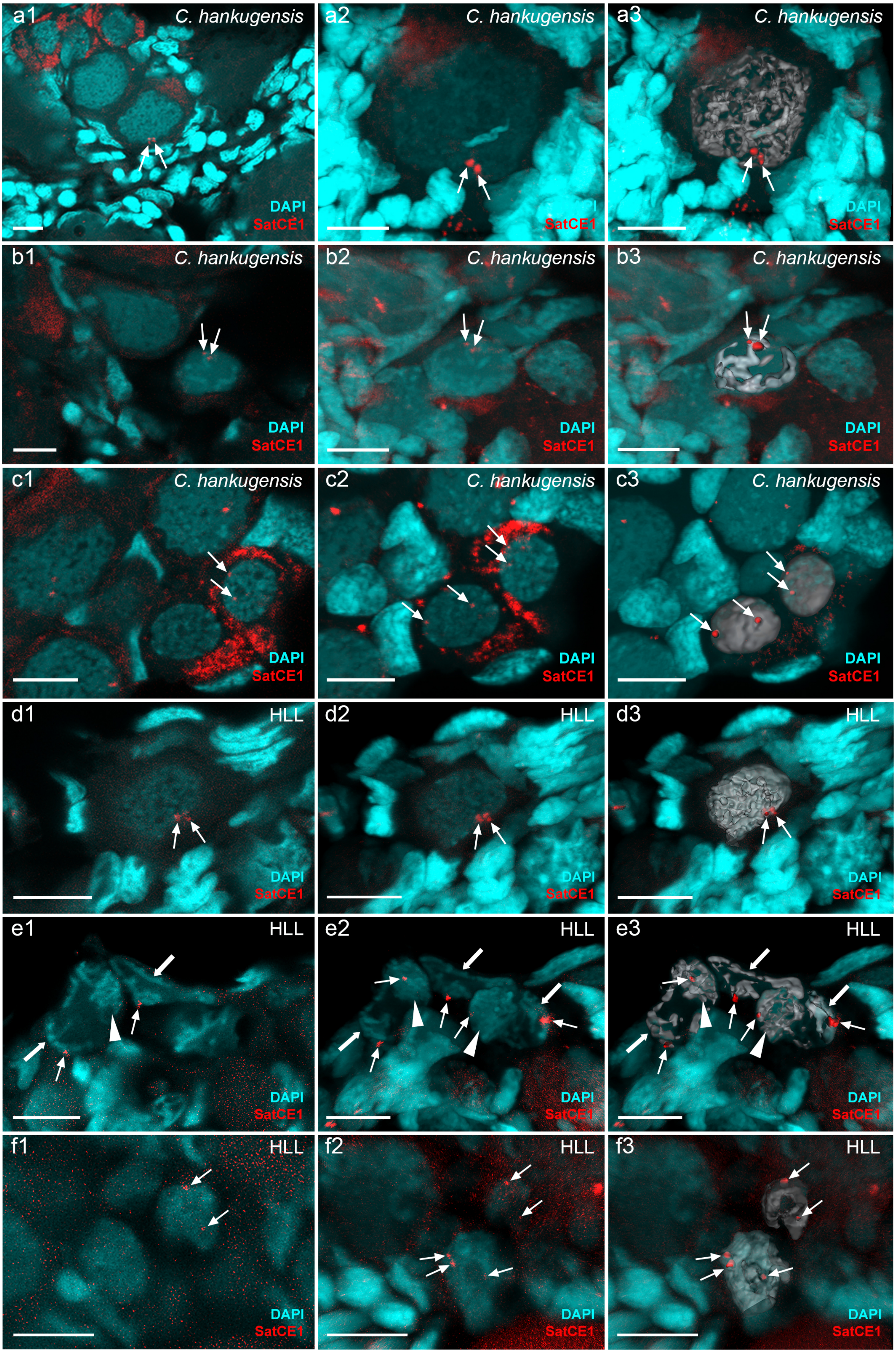

a1 C. hankugensis
a2 C. hankugensis
a3 C. hankugensis
b1 C. hankugensis
b2 C. hankugensis
b3 C. hankugensis
c1 C. hankugensis
c2 C. hankugensis
c3 C. hankugensis
d1 HLL
d2 HLL
d3 HLL
e1 HLL
e2 HLL
e3 HLL
f1 HLL
f2 HLL
f3 HLL
DAPI
SatCE1

Figure S8. Identification of ploidy of pachytene oocytes from triploid hybrids. Whole mount FISH with chromosome-specific marker SatCE1 (indicated by thin arrows) shown in green on bivalents and univalent; lateral elements of synaptonemal complexes (SYCP3 protein) are visualized by immunofluorescent staining. (a) pachytene oocytes with 24 bivalents and 25 univalents exhibit one signal (indicated by thin arrows) on bivalent (indicated by thick arrows) and one signal (indicated by thin arrows) on univalent (indicated by arrowheads). Pachytene oocytes with 24 bivalents (b) exhibit one signal (indicated by thin arrows) on bivalent (indicated by thick arrows). Pachytene oocytes with 25 univalents (c) exhibit one signal (indicated by thin arrows) on individual univalent (indicated by arrowheads). Scale bar = 10 µm.

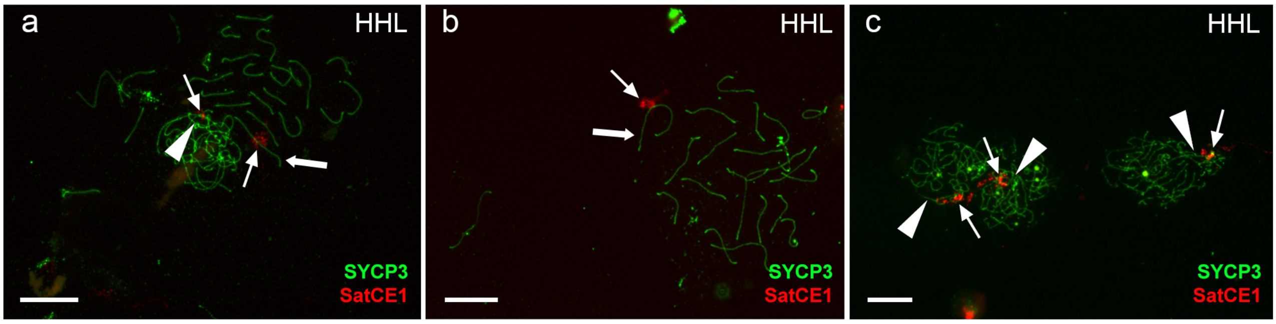
a
HHL
SYCP3
SatCE1
b
HHL
SYCP3
SatCE1
c
HHL
SYCP3
SatCE1

Figure S9. Map of South Korea (a) and enlarged map (b) with sampling localities of all studied fishes used in the study. 1. Deoksan-ri, Unbong-eup, Namwon-si, Jeollabuk-do (N 35.408076201033; E 127.518965284626), 2. Seocheon-ri, Unbong-eup, Namwon-si, Jeollabuk-do (N 35.4395259522794; E 127.524486803934), 3. Inwol-ri, Inwol-myeon, Namwon-si, Jeollabuk-do (N 35.45971510024; E 127.59280941924).

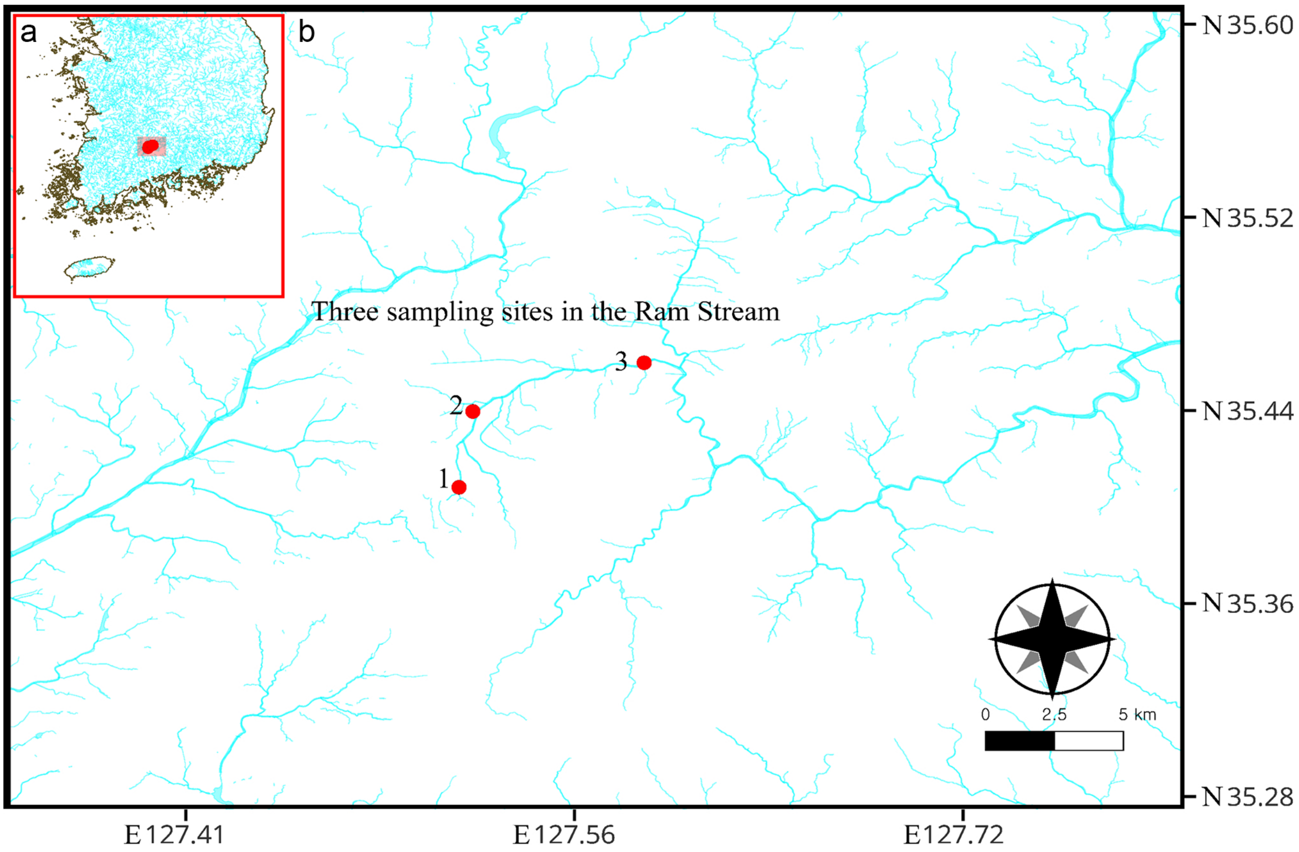
a
b
Three sampling sites in the Ram Stream
1
2
3
N 35.60
N 35.52
N 35.44
N 35.36
N 35.28
E 127.41
E 127.56
E 127.72
0
2.5
5 km

Supplementary Table S1. Summary of analysed meiotic cells and gonocytes for parental species, diploid and triploid hybrids

name	genotype	sex	pachytene			diplotene		examination of intact gonad	whole mount FISH with chromosome specific probe Sat CE1						
			univalents (1n)	bivalents (2n)	bi- and univalents (3n)	2n	4n		germ cells (2n)	germ cells (3n)	germ cells (4n)	pachytenes 1 signal (2n)	pachytenes 2 signals (3n or 4n)	diplotene 2 signals (2n)	diplotene 4 signals (4n)
Ko31519CH1M	HH	F	0	34	0										
Ko31519CH2M	HH	F				19	0								
Ko31519CH4M	HH	F				7	0	yes	4	0	0	18	0	3	0
Ko31519CH6F	HH	F						yes	23	0	0	98	0	161	0
Ko6619CH10F	HH	M	0	32	0			yes							
Ko6619CH12F	HH	M	0	9	0			yes							
Ko70922CH3F	HH	M	0	31	0										
Ko4619IL9F	LL	F	0	17											
Ko6619IL11	LL	Juv (F)	0	4				yes							
Ko6619IL12F	LL	F				9	0								
Ko_7619_IL13F	LL	F				4	0	yes							
Ko6619IL10M	LL	M	0	13				yes							
Ko809222n1F	HL	F	0	11	0	0		yes	106	0	2	0	34	0	3
Ko809222n2F	HL	F	0	2	0	0	38	yes	191	0	16	0	57	0	22
Ko609222n2F	HL	F						yes	49	0	3	0	38	0	6
Ko2619LH11F	HHL	F	0	1	41	10	0								
Ko3619LH12F	HHL	F	10	3	19	4	0								
Ko3619LH13F	HHL	F	0	0	8	9	0								
Ko4619LH15F	HHL	F	0	0	6	14	0								
Ko4619LH16F	HHL	F	0	0	3	24	0								
Ko4619LH17F	HHL	F	5	7	43										
Ko4619LH18F	HHL	F	29	12	72										
Ko6619LH19F	HHL	F	2	4	8	4	0								

name	geno type	sex	pachytene			diplotene		exami nation of intact gonad	whole mount FISH with chromosome specific probe Sat CE1						
			univalents (1n)	bivalents (2n)	bi- and univalents (3n)	2n	4n		germ cells (2n)	germ cells (3n)	germ cells (4n)	pachytenes 1 signal (2n)	pachytenes 2 signals (3n or 4n)	diplotene 2 signals (2n)	diplotene 4 signals (4n)
Ko6619LH20F	HHL	F	16	21	53										
Ko70922_3n1F	HHL	F	0	0	5	12	0	yes	3	41	0	1	34	89	0
Ko50922_3n1F	HHL	F						yes	2	36	0	1	5	14	0
Ko50922_3n2F	HLL	F						yes	1	10	0	1	4	61	0
Ko50922_3n3F	HLL	F						yes	12	41	0	24	86	142	0
Ko50922_3n4F	HLL	F						yes	4	36	0	2	6	63	0
Ko50922_3n5f	HHL	F						yes	7	86	0	9	45	96	0
Ko4619_3n14M	HHL	M			24			yes							
Ko50922_3n3M	HHL	M			33			yes							
Ko50922_3n4M	HHL	M						yes							
Ko90922_3n2M	HHL	M						yes							
