# Supplementary material for: A cyclical switch of gametogenic pathways in hybrids depends on the ploidy level
Source: Commun Biol. 2024 Apr 8;7:424. doi: 10.1038/s42003-024-05948-6 (PMC11001910; doi:10.1038/s42003-024-05948-6)
Supplement: Supplementary file 2 — Reporting summary [file 42003_2024_5948_MOESM2_ESM.pdf]

nature portfolio

Corresponding author(s): Dr. Karel Janko, Dr. Dmitrij Dedukh

Last updated by author(s): Feb 17, 2024

Reporting Summary

Nature Portfolio wishes to improve the reproducibility of the work that we publish. This form provides structure for consistency and transparency in reporting. For further information on Nature Portfolio policies, see our Editorial Policies and the Editorial Policy Checklist.

Statistics

For all statistical analyses, confirm that the following items are present in the figure legend, table legend, main text, or Methods section.

n/a	Confirmed	
☐	☒	The exact sample size (*n*) for each experimental group/condition, given as a discrete number and unit of measurement
☐	☒	A statement on whether measurements were taken from distinct samples or whether the same sample was measured repeatedly
☐	☒	The statistical test(s) used AND whether they are one- or two-sided<br>*Only common tests should be described solely by name; describe more complex techniques in the Methods section.*
☐	☒	A description of all covariates tested
☒	☐	A description of any assumptions or corrections, such as tests of normality and adjustment for multiple comparisons
☒	☐	A full description of the statistical parameters including central tendency (e.g. means) or other basic estimates (e.g. regression coefficient) AND variation (e.g. standard deviation) or associated estimates of uncertainty (e.g. confidence intervals)
☐	☒	For null hypothesis testing, the test statistic (e.g. *F*, *t*, *r*) with confidence intervals, effect sizes, degrees of freedom and *P* value noted<br>*Give P values as exact values whenever suitable.*
☒	☐	For Bayesian analysis, information on the choice of priors and Markov chain Monte Carlo settings
☒	☐	For hierarchical and complex designs, identification of the appropriate level for tests and full reporting of outcomes
☒	☐	Estimates of effect sizes (e.g. Cohen's *d*, Pearson's *r*), indicating how they were calculated

Our web collection on statistics for biologists contains articles on many of the points above.

Software and code

Policy information about availability of computer code

Data collection	No software was used
Data analysis	No software was used

For manuscripts utilizing custom algorithms or software that are central to the research but not yet described in published literature, software must be made available to editors and reviewers. We strongly encourage code deposition in a community repository (e.g. GitHub). See the Nature Portfolio guidelines for submitting code & software for further information.

Data

Policy information about availability of data

All manuscripts must include a data availability statement. This statement should provide the following information, where applicable:

- Accession codes, unique identifiers, or web links for publicly available datasets
- A description of any restrictions on data availability
- For clinical datasets or third party data, please ensure that the statement adheres to our policy

The authors state that all data necessary for confirming the conclusions presented in the article are represented fully within the article and its supplementary files.

April 2023

Research involving human participants, their data, or biological material

Policy information about studies with human participants or human data. See also policy information about sex, gender (identity/presentation), and sexual orientation and race, ethnicity and racism.

Reporting on sex and gender	not applicable
Reporting on race, ethnicity, or other socially relevant groupings	not applicable
Population characteristics	not applicable
Recruitment	not applicable
Ethics oversight	not applicable

Note that full information on the approval of the study protocol must also be provided in the manuscript.

Field-specific reporting

Please select the one below that is the best fit for your research. If you are not sure, read the appropriate sections before making your selection.

☒ Life sciences ☐ Behavioural & social sciences ☐ Ecological, evolutionary & environmental sciences

For a reference copy of the document with all sections, see nature.com/documents/nr-reporting-summary-flat.pdf

Life sciences study design

All studies must disclose on these points even when the disclosure is negative.

Sample size	We aimed to investigate gametogenesis in diploid and triploid hybrid females and males and compare it with gametogenesis of sexual males and females of both parental species. We collected samples of Cobitis hankugensis (4 females, 3 males) and Iksookimia longicorpa (4 females, 1 male) and their diploid (three females) and triploid (12 females with HHL genotype, 4 males with HHL genotype, 3 females with LLH genotype) hybrids. Sample size was not calculated as population size is not known. We observed similar pattern from three diploid hybrid females and 15 triploid hybrid females with around 2000 analysed germ cells and oocytes applying various method of analysis for the same individuals. It is important to note that investigated hybrids belonged to independent clonal strains. Hence observing exactly the same output in several independent individuals provides, in our opinion, convincing evidence in support of our claim.
Data exclusions	No data were excluded from the analysis. All counting were presented in the supplementary table 1 and throughout results and methods sections
Replication	All our analysed individuals with the same genotype and ploidy showed the similar results.
Randomization	Organisms were ranged according the genotype and ploidy level which was assessed by morphology and further confirmed by erythrocyte measurements and genetic analysis. Animals were occasionally collected from the field localities.
Blinding	As the aim of the study was to describe the gametogenic pathways in different forms of hybrids, the blinding method was not relevant to the study

Reporting for specific materials, systems and methods

We require information from authors about some types of materials, experimental systems and methods used in many studies. Here, indicate whether each material, system or method listed is relevant to your study. If you are not sure if a list item applies to your research, read the appropriate section before selecting a response.

Materials & experimental systems

n/a	Involved in the study
☐	☒ Antibodies
☒	☐ Eukaryotic cell lines
☒	☐ Palaeontology and archaeology
☐	☒ Animals and other organisms
☒	☐ Clinical data
☒	☐ Dual use research of concern
☒	☐ Plants

Methods

n/a	Involved in the study
☒	☐ ChIP-seq
☒	☐ Flow cytometry
☒	☐ MRI-based neuroimaging

Antibodies

Antibodies used	Lateral components of SCs were detected by rabbit polyclonal antibodies (ab15093, Abcam) against SYCP3 protein, the central component of SCs was detected by chicken polyclonal SYCP1 (gift from Prof. Sean Burgess; Blokhina, Y. P., Nguyen, A. D., Draper, B. W. & Burgess, S. M. The telomere bouquet is a hub where meiotic double-strand breaks, synapsis, and stable homolog juxtaposition are coordinated in the zebrafish, Danio rerio. PLOS Genetics 15, e1007730 (2019). Recombination loci were detected by mouse monoclonal antibodies against the MLH1 (ab14206, Abcam) proteins. Tubuline was detected by mouse polyclonal antibodies against alfa-tubulin (ab7291; Abcam).
Validation	All antibodies work in wide range of animals and previously were confirmed to work in loaches and other freshwater fishes, reptiles. As a confirmation please see:<br>Dedukh, D. et al. Achiasmatic meiosis in the unisexual Amazon molly, Poecilia formosa. Chromosome Research 30, 443–457 (2022).<br>Dedukh, D., Altmanová, M., Klíma, J. & Kratochvíl, L. Premeiotic endoreplication is essential for obligate parthenogenesis in geckos. Development 149, dev200345 (2022).<br>Dedukh, D. et al. Parthenogenesis as a solution to hybrid sterility: The mechanistic basis of meiotic distortions in clonal and sterile hybrids. Genetics 215, 975–987 (2020b).<br>Dedukh, D., Marta, A. & Janko, K. Challenges and costs of asexuality: Variation in premeiotic genome duplication in gynogenetic hybrids from Cobitis taenia complex. International Journal of Molecular Sciences 22, 12117 (2021).<br>Marta, A. et al. Genetic and karyotype divergence between parents affect clonality and sterility in hybrids. eLife 12:RP88366 (2023).<br>Blokhina, Y. P., Nguyen, A. D., Draper, B. W. & Burgess, S. M. The telomere bouquet is a hub where meiotic double-strand breaks, synapsis, and stable homolog juxtaposition are coordinated in the zebrafish, Danio rerio. PLOS Genetics 15, e1007730 (2019)

Animals and other research organisms

Policy information about studies involving animals; ARRIVE guidelines recommended for reporting animal research, and Sex and Gender in Research

Laboratory animals	The study did not involved laboratory animals
Wild animals	Analysis included two species of freshwater loach fishes (C. hankugensis and I. longicorpa), their natural diploid and triploid hybrids. Sampling was performed in the locality of the Ram Stream where pure parental individuals and diploid and triploid hybrids were collected. We collected samples of Cobitis hankugensis (4 females, 3 males) and Iksookimia longicorpa (4 females, 1 male) and their diploid (three females) and triploid (12 females with HHL genotype, 4 males with HHL genotype, 3 females with LLH genotype) hybrids from three sites along the Ram Stream in the province of Unbong-eup and Inwol-myeon Namwon-si Jeollabuk-do in Korea in field trip in 2019 and 2022. Collection sites: 1. Deoksan-ri, Unbong-eup, Namwon-si, Jeollabuk-do (N 35.408076201033; E 127.518965284626), 2. Seocheon-ri, Unbong-eup, Namwon-si, Jeollabuk-do (N 35.4395259522794; E 127.524486803934), 3. Inwol-ri, Inwol-myeon, Namwon-si, Jeollabuk-do (N 35.45971510024; E 127.59280941924). All animals except one C. hankugensis individual were adult; one C. hankugensis individual was juvenile female. Animals were collected by bottom net with mesh size 0,5 sm allowing to escape small organisms. Animals were placed into plastic tanks filled with water, aerated with air pumps and transferred to the Division of EcoScience, Ewha Womans University, Seoul, South Korea where they were processed. Analysis of meiotic chromosomes and different types of germ cells a well as the preparation of chromosomes from highly proliferating tissues such as kidneys requires gonadal tissue from alive animals. To dissect gonads and kidneys animals were sacrificed using an overdose of 2-phenoxyethanol anesthetics agent (Sigma). There is no alternative approach to obtain meiotic chromosomes as well as analyse germ cell line. Where it was possible we applied several methods of analysis for each particular individual. Ethical approval for the fish collection and experiments was obtained from the Institutional Animal Care and Use Committee (IACUC) at Ewha Womans University (IACUC permission no. 15-104).
Reporting on sex	We aimed to investigate gametogenesis in diploid and triploid hybrid females and males and compare it with gametogenesis of males and females of sexual species. We collected samples of Cobitis hankugensis (4 females, 3 males) and Iksookimia longicorpa (4 females, 1 male) and their diploid (three females) and triploid (12 females with HHL genotype, 4 males with HHL genotype, 3 females with LLH genotype) hybrids. As gametogenesis was not known, we analysed chromosomes during meiotic prophase for both males and females of sexual species. As we observed no difference between parental species, we did not increase the number of studied males of sexual species. Hybrid males are extremely rare in natural localities as they are sterile. We did not manage to collect diploid hybrid males but only females. For triploid hybrids we collected 12 females with HHL genotype and 4 males. Sex of individuals was identified initially by morphological features and further confirmed by gonadal morphology.
Field-collected samples	Animals were placed in several 50 liters (15 individuals per one aquarium) which were aerated with air pumps. Water temperature

Field-collected samples	was 23 degree with photo-period identical to natural conditions. Upon transportation animals were processed within 2 days for the meiotic and cytogenetic analysis.
Ethics oversight	Ethical approval for the fish collection and experiments was obtained from the Institutional Animal Care and Use Committee (IACUC) at Ewha Womans University (IACUC permission no. 15-104).

Note that full information on the approval of the study protocol must also be provided in the manuscript.

Plants

Seed stocks	not applicable
Novel plant genotypes	not applicable
Authentication	not applicable
